# Assessment of Lower Limb Muscle Strength and Power Using Hand-Held and Fixed Dynamometry: A Reliability and Validity Study

**DOI:** 10.1371/journal.pone.0140822

**Published:** 2015-10-28

**Authors:** Benjamin F. Mentiplay, Luke G. Perraton, Kelly J. Bower, Brooke Adair, Yong-Hao Pua, Gavin P. Williams, Rebekah McGaw, Ross A. Clark

**Affiliations:** 1 School of Exercise Science, Faculty of Health Sciences, Australian Catholic University, Melbourne, Australia; 2 School of Allied Health, Faculty of Health Sciences, Australian Catholic University, Melbourne, Australia; 3 Department of Physiotherapy, Singapore General Hospital, Singapore, Singapore; 4 Department of Physiotherapy, Epworth Healthcare, Melbourne, Australia; 5 School of Physiotherapy, Faculty of Medicine, Denistry and Health Sciences, The University of Melbourne, Melbourne, Australia; Purdue University, UNITED STATES

## Abstract

**Introduction:**

Hand-held dynamometry (HHD) has never previously been used to examine isometric muscle power. Rate of force development (RFD) is often used for muscle power assessment, however no consensus currently exists on the most appropriate method of calculation. The aim of this study was to examine the reliability of different algorithms for RFD calculation and to examine the intra-rater, inter-rater, and inter-device reliability of HHD as well as the concurrent validity of HHD for the assessment of isometric lower limb muscle strength and power.

**Methods:**

30 healthy young adults (age: 23±5yrs, male: 15) were assessed on two sessions. Isometric muscle strength and power were measured using peak force and RFD respectively using two HHDs (Lafayette Model-01165 and Hoggan micro*FET*2) and a criterion-reference KinCom dynamometer. Statistical analysis of reliability and validity comprised intraclass correlation coefficients (ICC), Pearson correlations, concordance correlations, standard error of measurement, and minimal detectable change.

**Results:**

Comparison of RFD methods revealed that a peak 200ms moving window algorithm provided optimal reliability results. Intra-rater, inter-rater, and inter-device reliability analysis of peak force and RFD revealed mostly good to excellent reliability (coefficients ≥ 0.70) for all muscle groups. Concurrent validity analysis showed moderate to excellent relationships between HHD and fixed dynamometry for the hip and knee (ICCs ≥ 0.70) for both peak force and RFD, with mostly poor to good results shown for the ankle muscles (ICCs = 0.31–0.79).

**Conclusions:**

Hand-held dynamometry has good to excellent reliability and validity for most measures of isometric lower limb strength and power in a healthy population, particularly for proximal muscle groups. To aid implementation we have created freely available software to extract these variables from data stored on the Lafayette device. Future research should examine the reliability and validity of these variables in clinical populations.

## Introduction

Muscular weakness, as a component of muscle function, is an impairment that is commonly observed in clinical populations and has been widely documented to impact upon physical function [1–4]. Two important components of muscle function are the peak force that a muscle group can produce (muscle strength) and how rapidly that force can be produced (muscle power) [3, 5]. The latter has previously been quantified by calculating the rate of force development (RFD), which is calculated by measuring the change in force over a certain time period (Δforce/Δtime), usually during an isometric contraction [5, 6]. The measure of RFD has important functional implications; sufficient RFD is necessary to perform quick and forceful muscle contractions, such as those observed during walking [5]. Previous literature indicates that reduced muscle power, often associated with aging, may contribute to reduced physical function and an increased risk of falls in a range of clinical populations [7–13]. As such, assessments of muscle power may be useful in clinical settings for identifying individuals at risk of falls and functional limitations.

Currently there are varying methods utilised to calculate RFD from isometric contractions. Commonly used methods involve calculating the change in force over the change in time with discrete time intervals from the onset of contraction to 30, 50 or 100ms [5, 14, 15]. However, onset of contraction has been defined in different ways including when the force reading exceeds a set threshold of either absolute values or percentages of a maximal voluntary contraction [5, 14, 16–18]. Other methods of calculating RFD involve examining successive time intervals (e.g. 5ms) during the initial rise in force to determine the peak RFD across the trial [19–21], or examining the RFD between percentages of the peak force (e.g. between 30 and 60% of peak force) [22]. There is currently no consensus as to which measure of RFD should be used in the assessment of muscle power.

The criterion-reference assessment of muscle strength and power involves fixed laboratory-based dynamometry. A limitation of laboratory-based dynamometers is they are expensive and cumbersome which precludes their use as a clinically-feasible device for routine patient assessment [23–25]. Other devices that can be used to assess dynamic muscle power include linear position transducers [26–28], the Nottingham power rig [28–30], and force plates [31, 32], however the cost, availability, time-consuming nature, and difficulty of implementation of such assessments may limit their use in clinical settings. Clinic-based assessment of muscle power is important to allow widespread access to testing and easily-interpreted results. Commonly used devices that measure isometric lower limb muscle strength include hand-held dynamometers (HHDs). These low-cost and portable devices are an appropriate and convenient method to assess muscle strength in a clinical setting due to their strong reliability and validity when compared with expensive laboratory-based dynamometers [23–25, 33, 34]. Previous psychometric literature assessing isometric lower limb strength using HHDs has focused predominantly on the knee extensors, with limited information on the validity of HHDs when assessing the muscle strength of other lower limb muscle groups [25, 34]. Additionally, the reliability and validity of HHDs for the assessment of isometric muscle power is currently unknown and warrants further investigation due to the importance of muscle power [30, 35].

The first aim of this study was to examine the reliability of different algorithms for assessment of RFD using fixed dynamometry. Secondly, this study aimed to determine the concurrent validity of two HHDs (Lafayette and Hoggan manufactured devices) compared to fixed dynamometry (KinCom) to assess isometric lower limb muscle strength and power using measures of peak force and RFD. Additionally, the intra-rater, inter-rater, and inter-device reliability of each device for the assessment of peak force and RFD was assessed. It was hypothesised that the HHDs would demonstrate good validity and reliability for the assessment of both muscle strength and power (intraclass correlation coefficients ≥0.75).

## Materials and Methods

### Participants

The isometric lower limb muscle strength and power of 30 healthy participants over the age of 18 was assessed. Participants were required to have no lower limb injury in the preceding two months or other comorbidities such as cardiovascular or respiratory conditions that could potentially impact on the assessment of muscle strength and power. This study used a concurrent validity, test-retest reliability design whereby participants attended two identical testing sessions. This study had approval from the Australian Catholic University Human Research Ethics Committee, where a convenience sample of participants were recruited. All participants gave written informed consent.

### Instrumentation

Two HHDs were used to assess lower limb strength and power: a Lafayette Manual Muscle Testing System Model-01165 (Lafayette Instrument Company, Lafayette IN, USA) and a Hoggan micro*FET*2 (Hoggan Scientific, LLC, Salt Lake City UT, USA). The two HHDs were left as purchased from each manufacturer with no additional padding secured to the devices. The approximate retail cost of the Lafayette device is US$1,200, with the Hoggan device costing approximately US$1,095 (plus US$495 for the software package). For determination of the validity of each HHD, participants were also assessed using a fixed, laboratory-based KinCom dynamometer (Chattex Corporation, Chattanooga TN, USA). Laboratory-based dynamometers can often cost in excess of US$50,000. All devices recorded force in kilograms and were calibrated once at the start of the study. Both assessors were male and were experienced at using such devices, with Assessor-A having one year experience using HHDs and Assessor-B having 10 years of clinical physiotherapy experience using HHDs.

### Procedure

**Fig 1:**
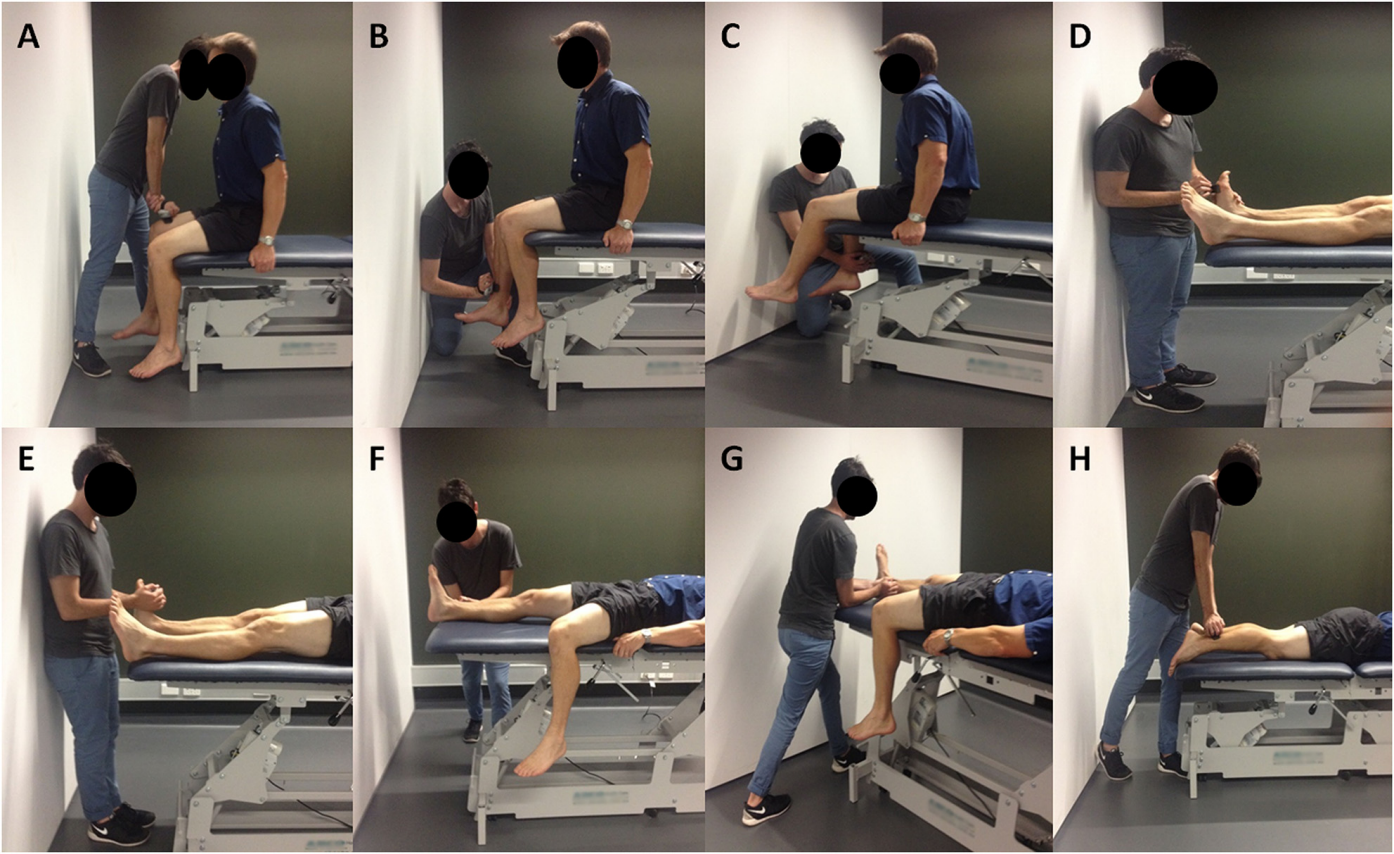
Testing positions for strength and power assessment. Note: Same positions were used on the fixed dynamometer. **(A)** Hip flexors with the participant seated and hips and knees flexed at 90°. Dynamometer placed on the anterior aspect of the thigh, proximal to the knee joint. **(B)** Knee extensors with the participant seated and hip and knees flexed at 90°. Dynamometer placed on the anterior aspect of the shank, proximal to the ankle joint. **(C)** Knee flexors with the participant seated and hips and knees flexed at 90°. Dynamometer placed on the posterior aspect of the shank, proximal to the ankle joint. **(D)** Ankle plantarflexors with the participant lying supine with the ankle in plantargrade and hips and knees extended. Dynamometer placed over the metatarsal heads on the sole of the foot. **(E)** Ankle dorsiflexors with the participant lying supine with the ankle relaxed and hips and knees extended. Dynamometer placed over the metatarsal heads on the dorsum of the foot. **(F)** Hip abductors with the participant lying supine and hips and knees extended. Dynamometer placed on the lateral aspect of the shank, proximal to the ankle joint. **(G)** Hip adductors with the participant lying supine and hips and knees extended. Dynamometer placed on the medial aspect of the shank, proximal to the ankle joint. **(H)** Hip extensors with the participant lying prone and hips and knees extended. Dynamometer placed on the posterior aspect of the shank, proximal to the ankle joint.

Currently there is no consensus on the most appropriate testing positions for HHD use, with a recent systematic review demonstrating a variety of methodologies used for lower limb assessment in previous research [25]. Based on prior research and our own pilot work of assessments in a variety of different positions, we implemented those shown in Fig 1. These testing positions have shown strong reliability for the measurement of isometric strength in previous studies for the hip [36], knee [37], and ankle [37] muscle groups.

Assessment of isometric muscle strength and power was performed with the participants in three positions (seated, supine, and prone); hip flexors, knee extensors, and knee flexors were assessed in a seated position; ankle plantarflexors, ankle dorsiflexors, hip abductors, and hip adductors in a supine position; hip extensors in a prone position. These positions were chosen to minimise changes in position by the participant to enhance the feasibility of testing in a clinical setting. All tests involved maximal voluntary isometric contractions. Assessment using the HHDs was conducted first. The order was randomised for assessor and HHD, however the order of the muscle groups tested was kept consistent as shown in Fig 1; for example if HHD1 was randomly assigned first, all seated muscle groups would be assessed, followed by HHD2 assessing seated muscle groups, with the same order of HHDs for supine and then prone muscle groups. Following a rest period of five minutes, the same protocol was repeated by the second assessor. During pilot testing, problems arose in the assessment of very strong muscle groups, namely the knee extensors and ankle plantarflexors. To assist the assessor in overcoming the force produced by the participant, the plinth was placed close to a wall, which aided the assessors in their resistance of the participants’ contractions for these two muscle groups (see Fig 1B and 1D).

Following HHD testing, the isometric strength and power of participants was then assessed using the KinCom dynamometer utilising the positions described for the HHDs. In order to minimise position changes and reduce time requirements, the order of muscles tested was different during the assessment with the KinCom dynamometer. The order for the KinCom was as follows: knee extensors, knee flexors, hip flexors, hip abductors, hip adductors, hip extensors, ankle plantarflexors, and ankle dorsifexors. Instructions provided to participants for all trials were ‘at the count of three, push/pull as hard and as fast as you can and hold that contraction until I say relax’. Each test lasted between three to five seconds and ended after a steady maximal force was produced by the participant. Participants were instructed to hold the side of the plinth for stabilization (see Fig 1). Constant verbal encouragement was provided throughout the testing. Only the right limb of each participant was assessed to reduce fatigue and the time demands of the testing session. A submaximal practice trial was given for each muscle group on both HHDs and the fixed dynamometer to ensure the participant understood the contraction required. Two trials were recorded for each muscle group, again to minimise the time requirements of testing.

### Data Analysis

A custom-written software program (LabVIEW 2009 National Instruments, Austin TX, USA) was made to analyse the data from the three devices using the following procedures. A zero-phase shift 10Hz lowpass 4^th^ order Butterworth filter was applied to data from each of the three devices. Due to the differing sampling rates between devices (Lafayette: stable 40Hz; Hoggan: unstable, approximately 100Hz; KinCom: stable 1000Hz), the data for the HHDs were resampled to a constant interval 1000Hz using cubic spline interpolation to allow for consistent and unbiased analysis. Strength was assessed by measuring peak force, which was determined by calculating the highest force value recorded in kilograms during both trials for each muscle group. Whilst normalisation to the length of the lever arm and body mass is crucial if comparing results from HHDs between participants, data from this study were not normalised in this way; our analysis of results was only performed within participants and therefore normalisation was redundant.

There is currently no consensus in the literature on the most appropriate measure of RFD [5, 19, 22]. Thus a comparison of the reliability of differing methodologies on the criterion-reference KinCom dynamometer was included in the current study. The analysis methods used included variants of three methods for the assessment of muscle power: 1) time to peak force, 2) calculating peak RFD between percentages of the peak force (5–95%, 10–90%, 15–85%, 20–80%, 25–75%, 30–70%, 35–65%, 40–60%), and, 3) examining successive time intervals (e.g. sample 1–11, 2–12, 3–13 etc.) during the initial rise in force to determine the peak RFD across the trial for time intervals of 10, 20, 50, 100, and 200ms. The methods differed in that the second method has a fixed position on the force trace but a variable time interval (i.e. it is always between the set force thresholds, but the duration shortens if the RFD is higher), whilst the third method has a fixed time interval but variable force position (i.e. the extracted data always has the same number of samples in it, but it could occur anywhere on the ascending slope of the force trace).

### Statistical Analysis

Data were assessed for normality using a Shapiro-Wilk test, with the data conforming to normal distribution. Descriptive statistics (mean and standard deviations) were used to describe participant demographics and anthropometrics and outcome measures of peak force and RFD. The first step in analysis was to calculate the reliability of different RFD algorithms from the fixed dynamometer, which was done through intraclass correlation coefficients (ICC_2,1_).

Assessment of intra-rater and inter-rater reliability was conducted using ICC_2,1_, standard error of measurement (SEM), and minimal detectable change (MDC) with 95% confidence intervals. The SEM and MDC were calculated using the formulas provided by Portney and Watkins [38] and expressed as percentages of the mean. The SEM was calculated by multiplying the standard deviation of the first session results by the square root of one minus the ICC ($SEM\;=\;{SD}_{1}\sqrt{1-ICC}$) [38]. The MDC was calculated using the following formula $MDC\;=\;z\;\times \;SEM\;\times \;\sqrt{2}$, where z = 1.96 (based on 95% confidence) and SEM is the standard error of measurement [38]. The association and agreement between assessors and devices, for inter-rater and inter-device reliability, were also measured using Pearson’s correlation (R) and concordance correlation coefficients (R_c_). The Pearson’s correlation coefficient assesses association irrespective of magnitude differences whereas the concordance coefficient assesses both association and deviations from the line of identity (y = x).

Analysis of concurrent validity was conducted by comparing results from the two HHDs to the gold standard laboratory-based KinCom using ICC_2,1_, R, and R_c_. Standard or regression-based (when proportional bias was detected) Bland-Altman plots with 95% limits of agreement [39] were calculated for all variables (see S1–S4 Files). Correlations of the difference between scores and the average scores were examined to detect a proportional bias (R>0.50), which indicated use of a regression-based Bland-Altman plot. Point estimates of the correlation and ICC values for reliability and validity analyses were based on those provided by Portney and Watkins [38] interpreted as excellent (≥0.90), good (0.75–0.89), moderate (0.50–0.74), or poor (<0.50).

## Results

A convenience sample of thirty participants (age: 22.87±5.08yrs, mass: 68.67±9.15kg, height: 172.85±9.11cm, male: 15) who were recruited through the University attended two testing sessions one week apart (mean: 7±2 days). One participant was unable to attend the second session. Further explanation of missing data is provided in S1–S4 Files and the full data set is in S5 File.

### Reliability of RFD measures

Measures of time to peak force and RFD that involved calculating the change in force over the change in time between percentages of the peak force (5–95%, 10–90%, 15–85%, 20–80%, 25–75%, 30–70%, 35–65%, 40–60%) revealed poor to moderate test re-test reliability (median ICC<0.85) across the majority of muscle groups on the fixed KinCom dynamometer (Table 1). Examination of the RFD measures calculated across successive time intervals (10, 20, 50, 100, and 200ms) showed good to excellent results for test re-test reliability on the KinCom dynamometer (median ICC≥0.91). The 200ms time interval method displayed the highest median reliability results (median ICC = 0.93) and no results lower than our threshold for good (≥0.75), and was therefore used for further analyses.

**Table 1:** Test-retest reliability (ICCs) of different rate of force development measures on the fixed KinCom dynamometer.

Musclegroups	Time to peak	Percentage of peak force measures								Successive time intervals				
		RFD (5–95)	RFD (10–90)	RFD (15–85)	RFD (20–80)	RFD (25–75)	RFD (30–70)	RFD (35–65)	RFD (40–60)	Peak RFD (10ms)	Peak RFD (20ms)	Peak RFD (50ms)	Peak RFD (100ms)	Peak RFD (200ms)
**ADF**	-0.93	0.24	0.49	0.71	0.7	0.65	0.63	0.63	0.62	0.64	0.65	0.62	0.72	0.77
**APF**	0.67	0.95	0.96	0.97	0.95	0.87	0.85	0.88	0.88	0.96	0.96	0.96	0.95	0.95
**HAB**	-0.47	-0.22	0.45	0.59	0.77	0.82	0.81	0.79	0.83	0.83	0.83	0.86	0.9	0.88
**HAD**	0.46	0.47	0.62	0.56	0.59	0.75	0.74	0.77	0.77	0.78	0.79	0.8	0.88	0.92
**HE**	0.40	0.41	0.17	0.26	0.54	0.57	0.84	0.79	0.85	0.91	0.91	0.91	0.91	0.87
**HF**	0.70	0.77	0.92	0.94	0.95	0.95	0.94	0.94	0.94	0.95	0.95	0.95	0.95	0.94
**KE**	0.75	0.82	0.83	0.91	0.91	0.92	0.91	0.9	0.9	0.97	0.97	0.97	0.97	0.98
**KF**	0.39	0.77	0.84	0.78	0.73	0.7	0.66	0.66	0.67	0.93	0.93	0.92	0.89	0.93
*Median*	0.43	0.62	0.73	0.75	0.75	0.79	0.83	0.79	0.84	0.92	0.92	0.92	0.91	0.93
*IQR*	0.18–0.68	0.37–0.78	0.48–0.86	0.58–0.92	0.67–0.92	0.69–0.88	0.72–0.87	0.74–0.89	0.75–0.89	0.82–0.95	0.82–0.95	0.85–0.95	0.89–0.95	0.88–0.94
*N = <0*.*75*	7	4	4	4	4	3	3	2	2	1	1	1	1	0

Abbreviations: RFD: rate of force development; ADF: ankle dorsiflexors; APF: ankle plantarflexors; HAB: hip abductors; HAD: hip adductors; HE: hip extensors; HF: hip flexors; KE: knee extensors; KF: knee flexors; IQR: interquartile range (25–75%); N = <0.75: number of muscle groups below the threshold of 0.75.

### Intra- and Inter-rater Reliability

The mean (standard deviation (SD)) and intra-rater reliability results for peak force and RFD are shown in Tables 2 and 3 respectively. Intra-rater reliability was good to excellent (ICC≥0.75) for all peak force measures with the exception of a moderate result for the ankle plantarflexors measured by Assessor-B using the Hoggan device (ICC = 0.74). Intra-rater reliability was also good to excellent for all RFD measures with the exception of the knee extensors measured by Assessor-A using the Hoggan device (ICC = 0.71), and measures of ankle dorsiflexors (ICC = 0.49), hip abductors (ICC = 0.74), and knee extensors (ICC = 0.71) using the Lafayette device by Assessor-B.

**Table 2:** Mean (SD) values and intra-rater reliability for each assessor on each device plus the KinCom for peak force (kg).

Muscle Groups	Intra-rater reliability	Assessor-A		Assessor-B		KinCom
		Lafayette	Hoggan	Lafayette	Hoggan	
**ADF**	Day 1—Mean (SD)	19.19 (4.92)	20.89 (3.64)	27.47 (5.96)	30.68 (6.89)	18.47 (6.87)
	Day 2—Mean (SD)	17.83 (4.35)	20.92 (4.11)	27.42 (5.85)	29.93 (5.49)	17.52 (6.30)
	ICC (95% CI)	0.89 (0.76,0.95)	0.87 (0.71,0.94)	0.88 (0.75,0.95)	0.87 (0.71,0.94)	0.78 (0.43,0.92)
	SEM (%)	8.62	6.20	7.39	8.13	17.45
	MDC (%)	23.89	17.19	20.47	22.52	48.36
**APF**	Day 1—Mean (SD)	51.00 (10.94)	48.06 (8.12)	52.29 (11.17)	51.16 (10.85)	91.02 (35.94)
	Day 2—Mean (SD)	50.42 (11.34)	47.83 (9.70)	51.95 (10.05)	51.30 (11.27)	83.16 (36.13)
	ICC (95% CI)	0.84 (0.66,0.93)	0.87 (0.70,0.95)	0.87 (0.72,0.94)	0.74 (0.38,0.89)	0.98 (0.95,0.99)
	SEM (%)	8.53	6.06	7.70	10.81	5.72
	MDC (%)	23.64	16.81	21.35	29.97	15.86
**HAB**	Day 1—Mean (SD)	13.85 (3.73)	13.23 (3.91)	13.06 (3.03)	13.38 (3.83)	11.91 (3.39)
	Day 2—Mean (SD)	13.01 (3.27)	12.77 (3.50)	12.46 (3.71	12.94 (3.74)	11.14 (3.45)
	ICC (95% CI)	0.87 (0.73,0.94)	0.94 (0.86,0.97)	0.92 (0.84,0.96)	0.95 (0.89,0.98)	0.95 (0.88,0.98)
	SEM (%)	9.59	7.30	6.43	6.53	6.17
	MDC (%)	26.59	20.23	17.82	18.11	17.10
**HAD**	Day 1—Mean (SD)	18.27 (6.31)	17.53 (5.81)	19.65 (6.91)	20.37 (7.27)	19.56 (5.91)
	Day 2—Mean (SD)	18.57 (6.27)	18.16 (5.84)	19.10 (7.45)	19.56 (6.99)	18.92 (6.81)
	ICC (95% CI)	0.96 (0.92,0.98)	0.97 (0.92,0.99)	0.97 (0.93,0.99)	0.97 (0.92,0.99)	0.98 (0.94–0.99)
	SEM (%)	6.82	5.74	6.09	6.68	4.48
	MDC (%)	18.89	15.91	16.87	18.51	12.42
**HE**	Day 1—Mean (SD)	23.01 (5.34)	23.60 (5.69)	25.25 (6.80)	24.41 (5.66)	25.82 (6.58)
	Day 2—Mean (SD)	23.45 (6.62)	23.34 (5.92)	25.16 (6.67)	24.31 (5.97)	25.43 (7.13)
	ICC (95% CI)	0.92 (0.82,0.96)	0.95 (0.90,0.98)	0.94 (0.86,0.97)	0.95 (0.88,0.98)	0.92 (0.81,0.97)
	SEM (%)	6.77	5.22	6.76	5.34	7.03
	MDC (%)	18.76	14.48	18.74	14.79	19.49
**HF**	Day 1—Mean (SD)	30.44 (7.84)	31.23 (7.82)	36.54 (8.23)	38.63 (8.26)	34.83 (10.48)
	Day 2—Mean (SD)	30.05 (6.53)	31.72 (7.81)	36.62 (6.74)	36.53 (7.50)	35.86 (9.73)
	ICC (95% CI)	0.94 (0.88,0.97)	0.95 (0.89,0.98)	0.93 (0.86,0.97)	0.85 (0.67,0.94)	0.95 (0.89,0.98)
	SEM (%)	6.15	5.43	5.83	8.17	6.45
	MDC (%)	17.05	15.05	16.16	22.65	17.89
**KE**	Day 1—Mean (SD)	44.27 (11.34)	50.41 (13.89)	43.92 (13.62)	47.70 (13.03)	63.54 (23.76)
	Day 2—Mean (SD)	41.51 (11.55)	46.07 (12.49)	42.66 (13.52)	46.13 (13.86)	58.66 (25.19)
	ICC (95% CI)	0.91 (0.80,0.96)	0.90 (0.76,0.96)	0.92 (0.83,0.96)	0.89 (0.76,0.95)	0.98 (0.94,0.99)
	SEM (%)	7.73	8.54	8.72	8.98	5.67
	MDC (%)	21.42	23.67	24.16	24.88	15.72
**KF**	Day 1—Mean (SD)	23.28 (5.74)	23.58 (6.19)	27.55 (9.15)	29.46 (7.69)	25.84 (7.28)
	Day 2—Mean (SD)	23.19 (5.25)	23.99 (4.84)	27.49 (7.90)	28.67 (7.45)	25.73 (7.35)
	ICC (95% CI)	0.92 (0.83,0.96)	0.89 (0.71,0.96)	0.94 (0.87,0.97)	0.96 (0.90,0.98)	0.94 (0.86,0.98)
	SEM (%)	6.93	8.59	8.07	5.29	6.67
	MDC (%)	19.21	23.81	22.36	14.66	18.48

Abbreviations: ADF: ankle dorsiflexors; APF: ankle plantarflexors; HAB: hip abductors; HAD: hip adductors; HE: hip extensors; HF: hip flexors; KE: knee extensors; KF: knee flexors; SD: standard deviation; CI: confidence intervals; SEM: standard error measurement (expressed as a percentage of the mean); MDC: minimal detectable change with 95% confidence intervals (expressed as a percentage of the mean). A description of missing data is outlined in S1–S4 Files.

**Table 3:** Mean (SD) values and intra-rater reliability for each assessor on each device plus the KinCom for rate of force development (kg/s).

Muscle Groups	Intra-rater reliability	Assessor-A		Assessor-B		KinCom
		Lafayette	Hoggan	Lafayette	Hoggan	
**ADF**	Day 1—Mean (SD)	35.55 (11.38)	46.17 (12.51)	53.06 (14.63)	71.28 (18.92)	67.74 (28.40)
	Day 2—Mean (SD)	32.90 (10.27)	45.46 (13.73)	53.34 (17.96)	68.26 (19.70)	64.59 (25.61)
	ICC (95% CI)	0.87 (0.72,0.94)	0.84 (0.63,0.93)	0.49 (-0.10,0.76)	0.75 (0.44,0.88)	0.77 (0.40,0.91)
	SEM (%)	11.63	11.01	19.69	13.38	20.24
	MDC (%)	32.24	30.52	54.57	37.09	56.10
**APF**	Day 1—Mean (SD)	111.31 (35.70)	125.40 (35.58)	118.54 (38.41)	144.89 (40.28)	230.81 (113.89)
	Day 2—Mean (SD)	107.63 (27.01)	119.24 (35.82)	113.41 (27.76)	143.09 (41.15)	216.40 (111.54)
	ICC (95% CI)	0.89 (0.76,0.95)	0.81 (0.55,0.92)	0.85 (0.67,0.93)	0.81 (0.56,0.92)	0.95 (0.88,0.98)
	SEM (%)	10.64	12.37	12.68	12.05	10.81
	MDC (%)	29.48	34.28	35.14	33.41	29.96
**HAB**	Day 1—Mean (SD)	30.49 (10.01)	34.80 (13.56)	30.08 (9.19)	37.78 (15.86)	37.75 (15.12)
	Day 2—Mean (SD)	28.80 (7.54)	33.51 (9.30)	29.16 (8.30)	36.71 (13.45)	34.35 (13.64)
	ICC (95% CI)	0.84 (0.66,0.93)	0.90 (0.77,0.95)	0.74 (0.44,0.88)	0.89 (0.76,0.95)	0.88 (0.71,0.95)
	SEM (%)	13.08	12.50	15.69	13.92	13.65
	MDC (%)	36.27	34.65	43.49	38.59	37.83
**HAD**	Day 1—Mean (SD)	39.97 (17.13)	43.42 (19.90)	44.73 (19.67)	58.55 (27.95)	58.23 (24.36)
	Day 2—Mean (SD)	39.33 (13.02)	46.55 (14.52)	43.54 (16.15)	55.74 (22.40)	54.76 (27.89)
	ICC (95% CI)	0.91 (0.80,0.96)	0.87 (0.64,0.95)	0.93 (0.84,0.97)	0.94 (0.86,0.98)	0.92 (0.78,0.97)
	SEM (%)	13.00	16.85	11.96	11.69	12.13
	MDC (%)	36.04	46.69	33.15	32.40	33.61
**HE**	Day 1—Mean (SD)	47.42 (15.08)	56.88 (20.79)	58.21 (17.55)	72.18 (25.61)	83.10 (29.19)
	Day 2—Mean (SD)	48.26 (14.57)	56.31 (14.84)	55.82 (15.43)	67.79 (17.93)	84.39 (28.50)
	ICC (95% CI)	0.91 (0.80,0.96)	0.86 (0.69,0.94)	0.87 (0.73,0.94)	0.89 (0.74,0.95)	0.87 (0.68,0.95)
	SEM (%)	9.70	13.58	10.71	11.82	12.62
	MDC (%)	26.88	37.64	29.67	32.77	34.98
**HF**	Day 1—Mean (SD)	67.45 (18.88)	88.05 (23.72)	84.78 (23.54)	112.95 (30.30)	147.38 (46.94)
	Day 2—Mean (SD)	65.82 (17.32)	89.84 (22.51)	82.34 (18.84)	104.49 (28.15)	152.80 (54.08)
	ICC (95% CI)	0.88 (0.75,0.94)	0.86 (0.66,0.94)	0.82 (0.62,0.92)	0.87 (0.71,0.95)	0.94 (0.85,0.98)
	SEM (%)	9.65	10.26	11.68	9.52	7.87
	MDC (%)	26.76	28.43	32.38	26.39	21.80
**KE**	Day 1—Mean (SD)	83.24 (27.78)	126.25 (55.88)	87.65 (24.45)	125.37 (43.44)	210.61 (91.22)
	Day 2—Mean (SD)	82.36 (27.09)	106.23 (34.03)	80.38 (25.90)	112.72 (36.38)	200.01 (86.90)
	ICC (95% CI)	0.84 (0.66,0.93)	0.71 (0.26,0.88)	0.71 (0.37,0.87)	0.77 (0.50,0.90)	0.98 (0.95,0.99)
	SEM (%)	13.18	24.04	15.02	16.55	5.81
	MDC (%)	36.54	66.63	41.64	45.86	16.11
**KF**	Day 1—Mean (SD)	42.87 (16.77)	52.07 (17.22)	53.92 (24.01)	77.15 (27.58)	90.55 (28.42)
	Day 2—Mean (SD)	38.86 (13.53)	49.83 (16.10)	52.47 (15.47)	70.63 (20.90)	92.74 (36.16)
	ICC (95% CI)	0.91 (0.80,0.96)	0.78 (0.38,0.92)	0.85 (0.69,0.93)	0.83 (0.56,0.94)	0.93 (0.82,0.97)
	SEM (%)	11.99	15.65	17.02	14.69	8.48
	MDC (%)	33.24	43.38	47.17	40.73	23.51

Abbreviations: ADF: ankle dorsiflexors; APF: ankle plantarflexors; HAB: hip abductors; HAD: hip adductors; HE: hip extensors; HF: hip flexors; KE: knee extensors; KF: knee flexors; SD: standard deviation; ICC: intraclass correlation coefficient; CI: confidence intervals; SEM: standard error measurement (expressed as a percentage of the mean); MDC: minimal detectable change with 95% confidence intervals (expressed as a percentage of the mean). A description of missing data is outlined in S1–S4 Files.

Inter-rater reliability results are displayed in Table 4. Inter-rater reliability was good to excellent for both peak force and RFD measures (ICC≥0.75) in all muscle groups except for peak force of the ankle dorsiflexors (ICC = 0.68) and ankle plantarflexors (ICC = 0.66) measured on the Hoggan device, and RFD of the ankle dorsiflexors measured on the Lafayette device (ICC = 0.70). The tables also show the intra- and inter-rater reliability results of the SEM and MDC calculations, expressed as a percentage of the mean.

**Table 4:** Inter-rater and inter-device reliability for the HHDs.

Muscle Groups		Inter-rater reliability				Inter-device reliability			
		Peak Force (kg)		RFD (kg/s)		Peak Force (kg)		RFD (kg/s)	
		Lafayette	Hoggan	Lafayette	Hoggan	Assessor-A	Assessor-B	Assessor-A	Assessor-B
**ADF**	ICC (95% CI)	0.77 (0.50,0.89)	0.68 (0.29,0.86)	0.70 (0.36,0.86)	0.75 (0.44,0.89)				
	SEM (%)	11.30	11.54	16.05	13.41				
	MDC (%)	22.15	22.63	31.46	26.28				
	R (95% CI)	0.59 (0.29,0.79)	0.61 (0.29,0.81)	0.52 (0.19,0.74)	0.68 (0.40,0.84)	0.79 (0.59,0.90)	0.84 (0.68,0.92)	0.85 (0.70,0.93)	0.73 (0.49,0.87)
	R_c_ (95% CI)	0.25 (0.09,0.40)	0.19 (0.06,0.32)	0.24 (0.06,0.41)	0.26 (0.10,0.40)	0.66 (0.44,0.81)	0.76 (0.57,0.87)	0.53 (0.34,0.68)	0.44 (0.23,0.61)
**APF**	ICC (95% CI)	0.81 (0.60,0.91)	0.66 (0.24,0.85)	0.90 (0.79,0.95)	0.83 (0.61,0.92)				
	SEM (%)	9.33	11.18	10.25	11.74				
	MDC (%)	18.29	21.91	20.08	23.01				
	R (95% CI)	0.66 (0.39,0.83)	0.47 (0.10,0.73)	0.78 (0.58,0.89)	0.71 (0.45,0.86)	0.85 (0.70,0.93)	0.75 (0.52,0.88)	0.86 (0.71,0.93)	0.78 (0.57,0.89)
	R_c_ (95% CI)	0.66 (0.40,0.83)	0.44 (0.10,0.69)	0.77 (0.56,0.88)	0.66 (0.40,0.82)	0.80 (0.64,0.89)	0.75 (0.52,0.88)	0.74 (0.55,0.85)	0.61 (0.39,0.77)
**HAB**	ICC (95% CI)	0.92 (0.84,0.96)	0.95 (0.89,0.98)	0.92 (0.82,0.96)	0.88 (0.73,0.94)				
	SEM (%)	6.92	6.51	9.24	14.27				
	MDC (%)	13.56	12.75	18.10	27.97				
	R (95% CI)	0.89 (0.78,0.95)	0.91 (0.81,0.96)	0.85 (0.71,0.93)	0.80 (0.61,0.90)	0.96 (0.92,0.98)	0.92 (0.83,0.96)	0.90 (0.79,0.95)	0.84 (0.69,0.92)
	R_c_ (95% CI)	0.84 (0.71,0.91)	0.91 (0.82,0.96)	0.85 (0.71,0.92)	0.78 (0.59,0.88)	0.96 (0.91,0.98)	0.89 (0.81,0.94)	0.80 (0.65,0.88)	0.64 (0.47,0.77)
**HAD**	ICC (95% CI)	0.98 (0.96,0.99)	0.95 (0.88,0.98)	0.92 (0.82,0.96)	0.91 (0.79,0.96)				
	SEM (%)	4.54	7.72	12.59	14.00				
	MDC (%)	8.90	15.12	24.68	27.44				
	R (95% CI)	0.96 (0.92,0.98)	0.92 (0.82,0.97)	0.82 (0.64,0.91)	0.84 (0.66,0.93)	0.95 (0.89,0.98)	0.96 (0.91,0.98)	0.84 (0.66,0.93)	0.91 (0.81,0.96)
	R_c_ (95% CI)	0.94 (0.88,0.97)	0.86 (0.73,0.93)	0.77 (0.59,0.88)	0.71 (0.50,0.84)	0.95 (0.88,0.98)	0.96 (0.91,0.98)	0.71 (0.53,0.83)	0.73 (0.56,0.84)
**HE**	ICC (95% CI)	0.92 (0.82,0.96)	0.95 (0.89,0.98)	0.89 (0.77,0.95)	0.86 (0.70,0.94)				
	SEM (%)	7.29	5.34	10.15	13.36				
	MDC (%)	14.29	10.46	19.90	26.18				
	R (95% CI)	0.87 (0.74,0.94)	0.90 (0.79,0.95)	0.83 (0.67,0.92)	0.79 (0.59,0.90)	0.96 (0.92,0.98)	0.93 (0.85,0.97)	0.77 (0.56,0.89)	0.89 (0.77,0.95)
	R_c_ (95% CI)	0.78 (0.63,0.88)	0.89 (0.77,0.95)	0.64 (0.44,0.78)	0.61 (0.39,0.76)	0.95 (0.89,0.98)	0.89 (0.79,0.94)	0.64 (0.42,0.78)	0.70 (0.52,0.82)
**HF**	ICC (95% CI)	0.93 (0.85,0.97)	0.92 (0.80,0.96)	0.85 (0.69,0.93)	0.87 (0.69,0.94)				
	SEM (%)	6.39	6.71	10.76	9.80				
	MDC (%)	12.53	13.15	21.08	19.21				
	R (95% CI)	0.86 (0.73,0.93)	0.84 (0.66,0.93)	0.83 (0.67,0.92)	0.75 (0.49,0.89)	0.91 (0.80,0.96)	0.82 (0.64,0.91)	0.85 (0.69,0.93)	0.81 (0.62,0.91)
	R_c_ (95% CI)	0.69 (0.50,0.81)	0.63 (0.41,0.79)	0.61 (0.42,0.75)	0.57 (0.32,0.75)	0.91 (0.81,0.96)	0.81 (0.62,0.91)	0.54 (0.34,0.69)	0.56 (0.35,0.71)
**KE**	ICC (95% CI)	0.89 (0.77,0.95)	0.90 (0.77,0.96)	0.80 (0.56,0.91)	0.75 (0.44,0.89)				
	SEM (%)	9.30	8.76	13.84	19.58				
	MDC (%)	18.23	17.18	27.12	38.37				
	R (95% CI)	0.86 (0.71,0.93)	0.82 (0.63,0.92)	0.61 (0.31,0.80)	0.56 (0.21,0.78)	0.93 (0.85,0.97)	0.89 (0.77,0.95)	0.41 (0.02,0.69)	0.57 (0.25,0.78)
	R_c_ (95% CI)	0.84 (0.70,0.92)	0.81 (0.62,0.91)	0.60 (0.30,0.79)	0.54 (0.22,0.75)	0.83 (0.70,0.91)	0.85 (0.72,0.93)	0.24 (0.02,0.44)	0.31 (0.11,0.49)
**KF**	ICC (95% CI)	0.82 (0.62,0.91)	0.92 (0.77,0.97)	0.81 (0.60,0.91)	0.82 (0.49,0.94)				
	SEM (%)	12.53	7.40	18.46	14.71				
	MDC (%)	24.56	14.51	36.18	28.83				
	R (95% CI)	0.78 (0.58,0.89)	0.84 (0.59,0.94)	0.69 (0.44,0.84)	0.71 (0.33.0.89)	0.95 (0.88,0.98)	0.85 (0.68,0.93)	0.90 (0.76,0.96)	0.88 (0.74,0.95)
	R_c_ (95% CI)	0.61 (0.41,0.76)	0.70 (0.39,0.87)	0.55 (0.32,0.72)	0.43 (0.13,0.65)	0.94 (0.85,0.97)	0.84 (0.67,0.92)	0.78 (0.58,0.89)	0.66 (0.46,0.80)

Abbreviations: RFD: rate of force development; ADF: ankle dorsiflexors; APF: ankle plantarflexors; HAB: hip abductors; HAD: hip adductors; HE: hip extensors; HF: hip flexors; KE: knee extensors; KF: knee flexors; ICC: intraclass correlation coefficient; CI: confidence intervals; SEM: standard error measurement (expressed as a percentage of the mean); MDC: minimal detectable change with 95% confidence intervals (expressed as a percentage of the mean); R: Pearson’s correlation coefficient; R_c_: concordance correlation coefficient. A description of missing data is outlined in S1–S4 Files.

### Inter-device Reliability

Analysis of inter-device results showed good to excellent correlations (R≥0.75) between results obtained on the Lafayette and Hoggan devices for all peak force measures (Table 4). Additionally, concordance correlations for peak force also showed good to excellent agreement (R_c_≥0.75) with the exception of ankle dorsiflexors (R_c_ = 0.66) measured by Assessor-A. Inter-device analysis of RFD measures showed good to excellent correlations (R≥0.75) for all muscle groups with the exception of the ankle dorsiflexors measured by Assessor-B (R = 0.73) and the knee extensors for Assessors-A and B (R = 0.41, 0.57 respectively). The majority of RFD concordance correlation results showed moderate to good agreement (see Table 4). Measures of RFD for the knee extensors showed poor agreement and moderate correlations between devices for both assessors.

### Concurrent Validity

Results from the validity analysis for peak force and RFD measures are shown in Table 5. Validity of peak force measures were good to excellent (ICC≥0.75) with the exception of most ankle results which demonstrated moderate validity; this included ankle dorsiflexors measured by Assessor-A on the Lafayette device (ICC = 0.62) and the Hoggan device (ICC = 0.51) and ankle plantarflexors measured by Assessor-A and B on the Lafayette device (ICC = 0.51, 0.54 respectively) and the Hoggan device (ICC = 0.47, 0.40 respectively). The validity of RFD measures were mixed, however all measures of the hip musculature demonstrated good to excellent validity (ICC≥0.75) except for the hip abductors measured by Assessor-B using the Lafayette device (ICC = 0.74). Ankle and knee RFD measures displayed mostly moderate to good validity. Results from the Bland-Altman plots are provided in S1–S4 Files.

**Table 5:** Concurrent validity analysis of the HHDs for assessment of peak force and RFD measures compared to the KinCom.

Muscle Groups	Validity	Peak Force (kg)				RFD (kg/s)			
		Assessor-A		Assessor-B		Assessor-A		Assessor-B	
		Lafayette	Hoggan	Lafayette	Hoggan	Lafayette	Hoggan	Lafayette	Hoggan
**ADF**	ICC (95% CI)	0.62 (0.15,0.83)	0.61 (0.09,0.83)	0.79 (0.52,0.91)	0.76 (0.44,0.90)	0.41 (-0.32,0.74)	0.40 (-0.36,0.74)	0.31 (-0.56,0.70)	0.72 (0.35,0.88)
	R (95% CI)	0.46 (0.09,0.72)	0.49 (0.11,0.75)	0.66 (0.36,0.84)	0.61 (0.28,0.81)	0.35 (-0.04,0.65)	0.34 (-0.06,0.65)	0.23 (-0.18,0.57)	0.60 (0.27,0.80)
	R_c_ (95% CI)	0.44 (0.10,0.70)	0.39 (0.09,0.63)	0.30 (0.11,0.46)	0.22 (0.06,0.36)	0.11 (-0.02,0.24)	0.17 (-0.04,0.36)	0.16 (-0.12,0.41)	0.54 (0.24,0.75)
**APF**	ICC (95% CI)	0.51 (-0.12,0.78)	0.47 (-0.25,0.78)	0.54 (-0.14,0.81)	0.40 (-0.42,0.74)	0.73 (0.38,0.88)	0.70 (0.31,0.87)	0.70 (0.32,0.87)	0.54 (-0.06,0.80)
	R (95% CI)	0.49 (0.12,0.74)	0.59 (0.24,0.81)	0.51 (0.14,0.75)	0.41 (0.00,0.70)	0.73 (0.47,0.87)	0.69 (0.40,0.86)	0.67 (0.37,0.84)	0.44 (0.05,0.72)
	R_c_ (95% CI)	0.16 (0.02,0.30)	0.13 (0.03,0.23)	0.17 (0.03,0.30)	0.11 (-0.01,0.22)	0.24 (0.10,0.38)	0.30 (0.12,0.46)	0.25 (0.09,0.39)	0.23 (0.01,0.43)
**HAB**	ICC (95% CI)	0.88 (0.74,0.95)	0.89 (0.75,0.95)	0.91 (0.80,0.96)	0.91 (0.79,0.96)	0.82 (0.60,0.92)	0.82 (0.59,0.92)	0.74 (0.42,0.88)	0.88 (0.74,0.95)
	R (95% CI)	0.79 (0.58,0.90)	0.80 (0.59,0.91)	0.85 (0.69,0.93)	0.83 (0.65,0.92)	0.76 (0.53,0.89)	0.70 (0.42,0.86)	0.66 (0.37,0.83)	0.79 (0.58,0.90)
	R_c_ (95% CI)	0.66 (0.43,0.81)	0.75 (0.52,0.88)	0.80 (0.63,0.90)	0.77 (0.57,0.89)	0.63 (0.40,0.78)	0.70 (0.43,0.85)	0.52 (0.26,0.70)	0.79 (0.59,0.90)
**HAD**	ICC (95% CI)	0.95 (0.87,0.98)	0.94 (0.84,0.98)	0.95 (0.89,0.98)	0.94 (0.85,0.98)	0.86 (0.68,0.94)	0.92 (0.80,0.97)	0.92 (0.82,0.97)	0.94 (0.87,0.98)
	R (95% CI)	0.90 (0.78,0.96)	0.90 (0.76,0.96)	0.91 (0.80,0.96)	0.89 (0.75,0.95)	0.82 (0.62,0.92)	0.87 (0.70,0.95)	0.88 (0.74,0.95)	0.90 (0.78,0.96)
	R_c_ (95% CI)	0.89 (0.76,0.95)	0.85 (0.67,0.93)	0.91 (0.80,0.96)	0.89 (0.76,0.95)	0.58 (0.36,0.74)	0.79 (0.57,0.90)	0.74 (0.55,0.86)	0.89 (0.76,0.95)
**HE**	ICC (95% CI)	0.88 (0.72,0.95)	0.90 (0.76,0.95)	0.94 (0.85,0.97)	0.93 (0.85,0.97)	0.76 (0.46,0.90)	0.88 (0.73,0.95)	0.87 (0.69,0.94)	0.88 (0.72,0.95)
	R (95% CI)	0.80 (0.59,0.91)	0.82 (0.62,0.92)	0.88 (0.74,0.95)	0.89 (0.76,0.95)	0.76 (0.52,0.89)	0.84 (0.67,0.93)	0.83 (0.65,0.92)	0.80 (0.59,0.91)
	R_c_ (95% CI)	0.72 (0.49,0.85)	0.77 (0.57,0.89)	0.88 (0.74,0.94)	0.86 (0.71,0.93)	0.28 (0.13,0.42)	0.52 (0.32,0.68)	0.52 (0.31,0.67)	0.74 (0.51,0.87)
**HF**	ICC (95% CI)	0.94 (0.87,0.97)	0.94 (0.85,0.97)	0.94 (0.86,0.97)	0.92 (0.82,0.97)	0.77 (0.50,0.90)	0.78 (0.49,0.91)	0.80 (0.56,0.91)	0.92 (0.82,0.97)
	R (95% CI)	0.92 (0.83,0.96)	0.90 (0.77,0.96)	0.90 (0.79,0.95)	0.88 (0.74,0.95)	0.88 (0.75,0.95)	0.77 (0.53,0.90)	0.79 (0.58,0.90)	0.95 (0.89,0.98)
	R_c_ (95% CI)	0.80 (0.65,0.89)	0.84 (0.68,0.92)	0.87 (0.76,0.93)	0.81 (0.64,0.91)	0.19 (0.09,0.28)	0.30 (0.13,0.45)	0.28 (0.14,0.42)	0.61 (0.44,0.74)
**KE**	ICC (95% CI)	0.82 (0.58,0.92)	0.90 (0.76,0.96)	0.92 (0.82,0.97)	0.88 (0.72,0.95)	0.40 (-0.37,0.74)	0.82 (0.58,0.92)	0.63 (0.16,0.84)	0.67 (0.24,0.85)
	R (95% CI)	0.82 (0.63,0.92)	0.87 (0.71,0.94)	0.90 (0.78,0.96)	0.86 (0.70,0.94)	0.36 (-0.04,0.66)	0.72 (0.45,0.87)	0.68 (0.39,0.85)	0.57 (0.23,0.79)
	R_c_ (95% CI)	0.48 (0.28,0.64)	0.71 (0.51,0.84)	0.61 (0.42,0.75)	0.62 (0.42,0.77)	0.07 (-0.01,0.15)	0.38 (0.17,0.56)	0.13 (0.04,0.22)	0.25 (0.07,0.41)
**KF**	ICC (95% CI)	0.80 (0.55,0.91)	0.79 (0.39,0.93)	0.85 (0.67,0.93)	0.87 (0.66,0.95)	0.72 (0.38,0.88)	0.79 (0.41,0.92)	0.84 (0.64,0.93)	0.84 (0.60,0.93)
	R (95% CI)	0.68 (0.40,0.84)	0.66 (0.25,0.87)	0.76 (0.53,0.89)	0.76 (0.48,0.90)	0.65 (0.35,0.83)	0.73 (0.39,0.90)	0.73 (0.48,0.87)	0.72 (0.42,0.88)
	R_c_ (95% CI)	0.64 (0.37,0.81)	0.65 (0.25,0.86)	0.72 (0.49,0.85)	0.73 (0.44,0.88)	0.18 (0.06,0.30)	0.29 (0.09,0.48)	0.36 (0.17,0.52)	0.58 (0.29,0.77)

Abbreviations: RFD: rate of force development; ADF: ankle dorsiflexors; APF: ankle plantarflexors; HAB: hip abductors; HAD: hip adductors; HE: hip extensors; HF: hip flexors; KE: knee extensors; KF: knee flexors; ICC: intraclass correlation coefficient; CI: confidence intervals; R: Pearson’s correlation coefficient; R_c_: concordance correlation coefficient. A description of missing data is outlined in S1–S4 Files.

## Discussion

Hand-held dynamometry demonstrated good to excellent intra- and inter-rater reliability for the assessment of isometric lower limb muscle strength and power in a healthy population. Comparison of the HHDs to a laboratory-based dynamometer showed moderate to excellent concurrent validity for both measures of isometric lower limb strength and power. To the authors’ knowledge, this is the first study to evaluate the intra- and inter-rater reliability and validity of HHDs for assessing muscle strength in all major muscles of the lower limbs with a greater than poor sample size based on the COSMIN checklist [40], and the first to use HHDs to assess muscle power. These low-cost, portable, and easy-to-use devices have previously shown excellent results for use as a clinically-feasible alternative to laboratory-based dynamometry for the assessment of isometric muscle strength. The results from the current study indicate promise for HHDs in the assessment of isometric muscle power.

Previous literature has focussed primarily on the assessment and treatment of muscle strength in various clinical populations; however, muscle power is another important consideration. Evidence indicates that in an elderly population, measures of muscle power are more strongly associated with self-reported function and incidence of falls than muscle strength [30, 35]. As such, knowledge of both muscle strength and power may be of use to clinicians when assessing and treating their patients. The HHD results for both peak force and RFD can be obtained from the same trial using the same methodology, adding to the feasibility of using this device in a clinical setting for patient assessment. A potential limiting factor is the lack of widely available software to extract the RFD data. For this reason we have created a freely available software program (available at http://www.instrumentedmovement.com) which allows the user to obtain the 200ms rolling window RFD from data stored on a Lafayette device. A software program for data from a Hoggan device is not available on the website due to the additional cost of purchasing the data recording software for this device and issues experienced during testing with saving recorded data (See S1–S5 Files).

The inter-rater reliability was good to excellent for both peak force and RFD using both HHD devices. Nonetheless, agreement between assessors ranged from moderate to excellent for peak force and RFD, suggesting that although results between assessors are comparable, the results are not interchangeable. Previous research has found mixed inter-rater reliability in a range of populations for the assessment of muscle strength [34, 41–45]. Both assessors in the current study were male, with differing levels of experience. Prior research has compared reliability results for peak force analysis using a Hoggan micro*FET*2 HHD and found similar reliability between male and female assessors with varying levels of experience, height, and weight [46]. Previous studies have commented on the influence that assessor strength may have on HHD testing [34, 47]. In our experience, sufficient strength levels are required to control the movement of the patient, after which the technique of the assessor is likely to be just as important for obtaining valid results. During testing assessors should have a wide base of support, use their own body mass to lean into the participant and keep arms tucked in towards their body.

Closer examination of the results from each lower limb muscle group revealed that the hip musculature showed the strongest reliability and validity for measures of peak force and RFD. Previous research examining peak force has also found similar results for the assessment of hip strength using HHD in a range of populations [36, 37, 48–50]. Assessment of the ankle muscles demonstrated good to excellent reliability however validity was lower than expected. Previous research in a healthy population has also shown poor validity of HHD measures of plantarflexor strength in comparison with the KinCom [51]. Assessment of the ankle muscle groups is important, because the ankle plantarflexors have a primary role in power generation during walking [52] and the dorsiflexors are the lower body muscles most strongly associated with gait speed in people living with stroke [53]. Our mixed validity results in the current study and previous research [51] may have been caused by the ankle plantar/dorsiflexor attachment used on the KinCom. Participants reported difficulty in using the attachment, especially for ankle dorsiflexion, due to the lack of stabilisation that the attachment provides. Moreover, the ankle attachment does not fit tightly within the load cell, which may have resulted in measurement error. Similar comments have been made previously using the ankle inversion/eversion attachment on the KinCom [54]. Assessment of peak force of the knee extensors and flexors demonstrated good to excellent reliability and validity however validity of RFD measures for the knee extensors using both HHDs ranged from moderate to good. This may have been due to the higher levels of force and power generated in the knee extensors, leading to the assessors having difficulty in stabilising the HHD during the initial rapid rise in force, consequently impacting on measures of RFD. Therefore, if the knee extensors are the primary muscle of interest it may be necessary to consider external bracing during power assessment. Analysis of the SEM for intra-rater reliability for each device showed small percentages of the mean indicating low measurement error, with RFD higher than peak force values (<10% for peak force except one measure and <20% for RFD except for one measure). The MDC results were also higher for RFD measures compared with peak force (<50% of the mean for RFD except two measures and <25% for peak force except for two measures). Analysis of the MDC for HHD measures of muscle strength and power may prove more informative in clinical populations than the healthy participants used in the current study.

The measurement of RFD has been widely used previously with a lack of consensus of which method is appropriate. After a comparison of various techniques for assessing RFD that were applicable to a HHD, this study utilised a peak 200ms iterative windowed time period method to determine peak RFD. Previous work has commented on the arbitrary nature of determining onset of contraction for calculation of RFD [55]. As such, this study did not determine the onset of contraction; rather RFD was identified using algorithms calculated from the first sample recorded to determine the peak RFD across the trial. This method ignores any erroneous recordings made by placing the HHD on the lower limb and as such the calculation of RFD will not include these initial recordings. Whilst increasing the duration of the window in the calculation of RFD may produce higher reliability results, a longer time window may include unwanted plateaus. We found the 200ms successive time window analysis technique to be robust to different sources of error during testing, however further research is needed in clinical populations to verify the findings of the current study.

Comparison of the Hoggan and Lafayette HHDs used in this study revealed no apparent differences between the devices in their reliability or validity for either measure of peak force or RFD. The inter-device reliability indicated that peak force results are interchangeable between the two different HHDs. Caution is necessary if interchanging RFD results between devices, as this study demonstrated mixed agreement between HHDs for measures of RFD. Additionally, both HHDs demonstrated mixed agreement with the KinCom for measures of peak force and RFD. The lack of agreement between devices for measures of RFD may be due to the different sampling rates employed by each device. Based on the results of the current study, there can be no recommendation as to which HHD should be used, with both devices demonstrating similar reliability and validity. One consideration for the future development of HHDs is the real-time calculation of RFD. Calculation of RFD on both of the devices chosen for this study currently requires post-testing analysis. The Hoggan device needs to be wirelessly connected to a computer during testing; with the software interface occasionally losing recorded data during collection (see S1–S5 Files). The Lafayette device stores raw data within the device, which can be downloaded to a laptop for analysis. After further testing, manufacturers should consider including RFD as an automated output on their device.

### Study Limitations

The sample used in this study was a group of young, healthy, and physically active individuals. Even with the assessors bracing against a wall, the assessment of the knee extensors and ankle plantarflexors could not be completed for one male participant. Additionally, as can be seen in Tables 2 and 3, these two muscle groups recorded much higher strength and power values on the fixed dynamometer across all participants. It is likely that the assessment of muscle strength and power would be easier in those with muscle weakness, such as the elderly or those with neurological impairments. The findings of this study may therefore not be directly generalisable to some clinical populations. A recent review demonstrated that the reliability of HHDs is generally lower in healthy populations compared to clinical populations [34]. This could be due to the difficulties when testing stronger participants or lower inter-subject variability in healthy populations, compared to clinical. Nonetheless, the inclusion of healthy individuals does not discount the importance of our study, as normative data is required, albeit not normalised to body mass, to allow comparison with healthy populations and therefore establishing reliability and validity in this group was considered essential.

## Conclusions

Hand-held dynamometry is a reliable and valid tool for the assessment of isometric lower limb muscle strength and power, which may be valuable information particularly in clinical populations with gait impairments. Assessment of muscle strength and power in clinical populations using HHDs is warranted to determine the psychometric properties of these devices.

## Competing Interests

The authors have declared that no competing interests exist.

## Supporting Information

S1 File Bland-Altman Plots indicating the validity of the Lafayette HHD in comparison with the KinCom for analysis of peak force (kg). (PDF)

S2 File Bland-Altman Plots indicating the validity of the Lafayette HHD in comparison with the KinCom for analysis of rate of force development (kg/s). (PDF)

S3 File Bland-Altman Plots indicating the validity of the Hoggan HHD in comparison with the KinCom for analysis of peak force (kg). (PDF)

S4 File Bland-Altman Plots indicating the validity of the Hoggan HHD in comparison with the KinCom for analysis of rate of force development (kg/s). (PDF)

S5 File Study outcome measures. (XLSX)
