# Supplementary material for: Assessment of Lower Limb Muscle Strength and Power Using Hand-Held and Fixed Dynamometry: A Reliability and Validity Study
Source: PLoS One. 2015 Oct 28;10(10):e0140822. doi: 10.1371/journal.pone.0140822 (PMC4624940; doi:10.1371/journal.pone.0140822)

Assessment of lower limb muscle strength and power using hand-held and fixed dynamometry: a reliability and validity study

S1 Appendix – Bland-Altman Plots indicating the validity of the Lafayette HHD in comparison with the KinCom for analysis of peak force (kg)

Note: Not all reliability and validity analyses include 30 data sets. Some participants mentioned soreness in some muscle groups unrelated to the testing procedures, and as such those sore muscles were not tested. The knee extensors and ankle plantarflexors of one participant were unable to be tested due to high strength and power levels of the participant. One participant was unable to attend the second testing session. The KinCom was unable to be used at all for four participants as the device was being repaired and five participants only had one session of KinCom data collection. On many occasions the Hoggan software failed to save the raw data during testing, resulting in fewer data sets for all analyses involving the Hoggan device. The parentheses prior to each figure details the number of participants that were used for analysis.

Assessor-A ankle dorsiflexors:

(n: validity 26; Lafayette intra-rater reliability 29; KinCom reliability 19; inter-rater reliability 29; inter-device reliability 27)

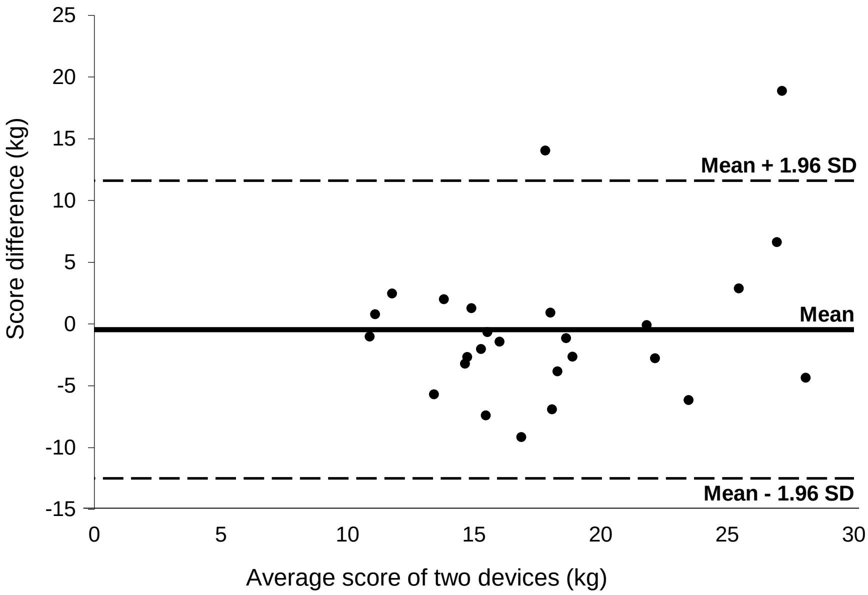

Assessor-A ankle plantarflexors:

(n: validity 25; Lafayette intra-rater reliability 28; KinCom reliability 20; inter-rater reliability 29; inter-device reliability 27)

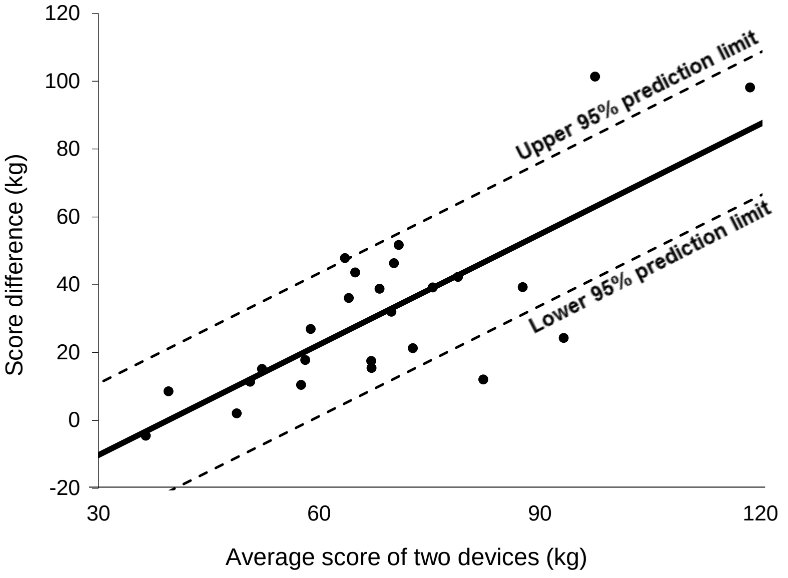

R = 0.77**;** R^2 = 0.59**; Slope =** 1.08**; Intercept =** -42.68

Assessor-A hip abductors:

(n: validity 26; Lafayette intra-rater reliability 29; KinCom reliability 20; inter-rater reliability 30; inter-device reliability 28)

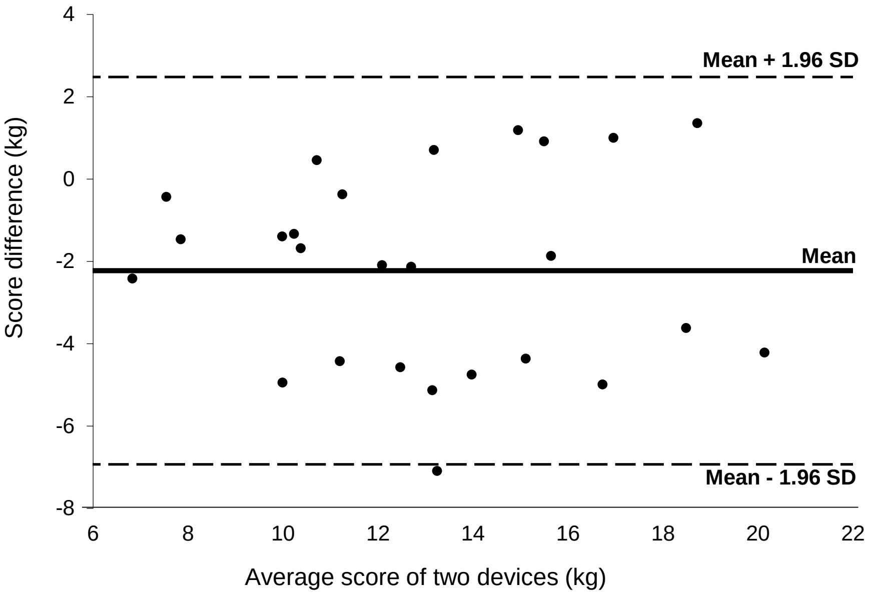

Assessor-A hip adductors:

(n: validity 24; Lafayette intra-rater reliability 27; KinCom reliability 18; inter-rater reliability 28; inter-device reliability 24)

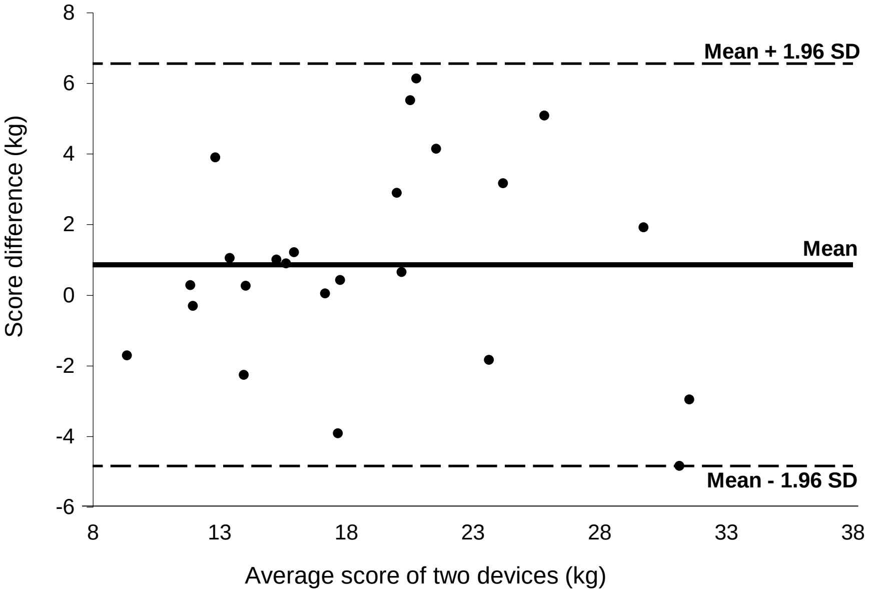

Assessor-A hip extensors:

(n: validity 25; Lafayette intra-rater reliability 28; KinCom reliability 20; inter-rater reliability 30; inter-device reliability 29)

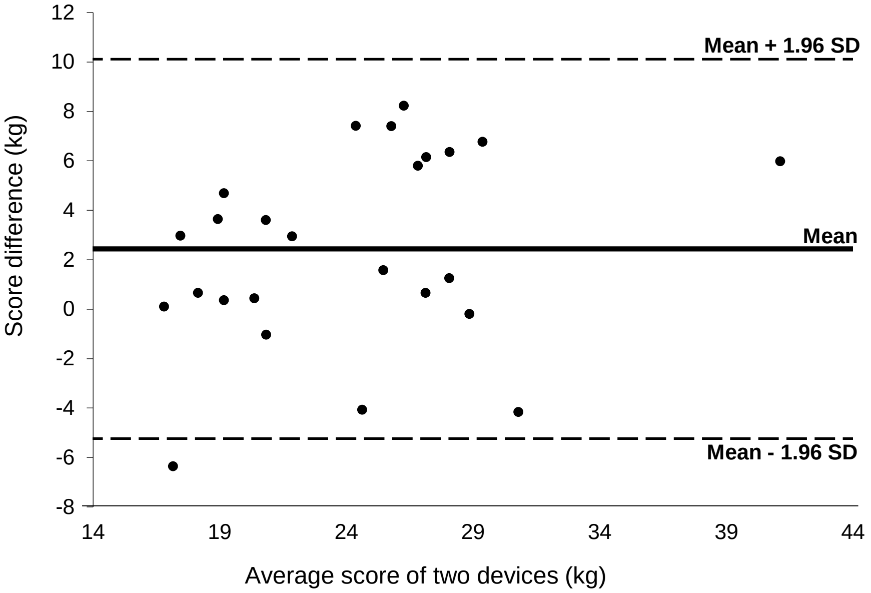

Assessor-A hip flexors:

(n: validity 26; Lafayette intra-rater reliability 29; KinCom reliability 20; inter-rater reliability 30; inter-device reliability 25)

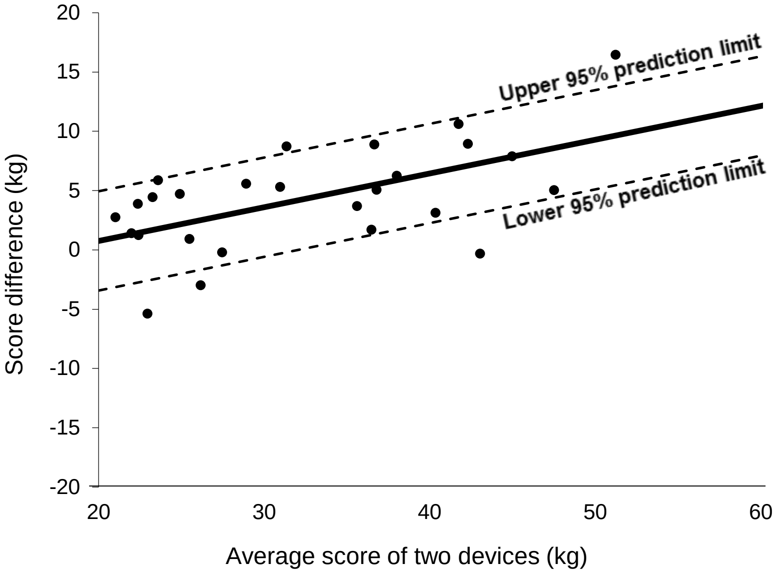

R = 0.58**; R^2 =** 0.33**; Slope =** 0.28**; Intercept =** -4.92

Assessor-A knee extensors:

(n: validity 25; Lafayette intra-rater reliability 27; KinCom reliability 20; inter-rater reliability 27; inter-device reliability 25)

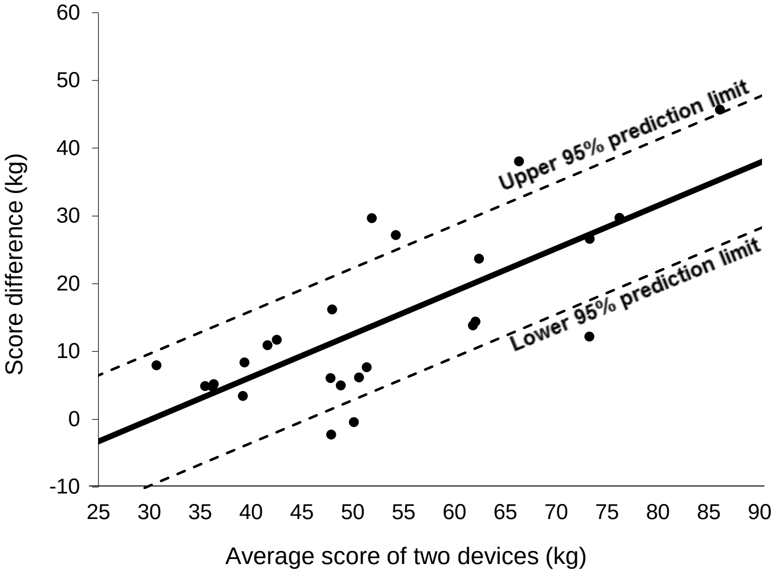

R = 0.74**; R^2 =** 0.54**; Slope =** 0.63**; Intercept =** -19.04

Assessor-A knee flexors:

(n: validity 26; Lafayette intra-rater reliability 29; KinCom reliability 20; inter-rater reliability 30; inter-device reliability 20)

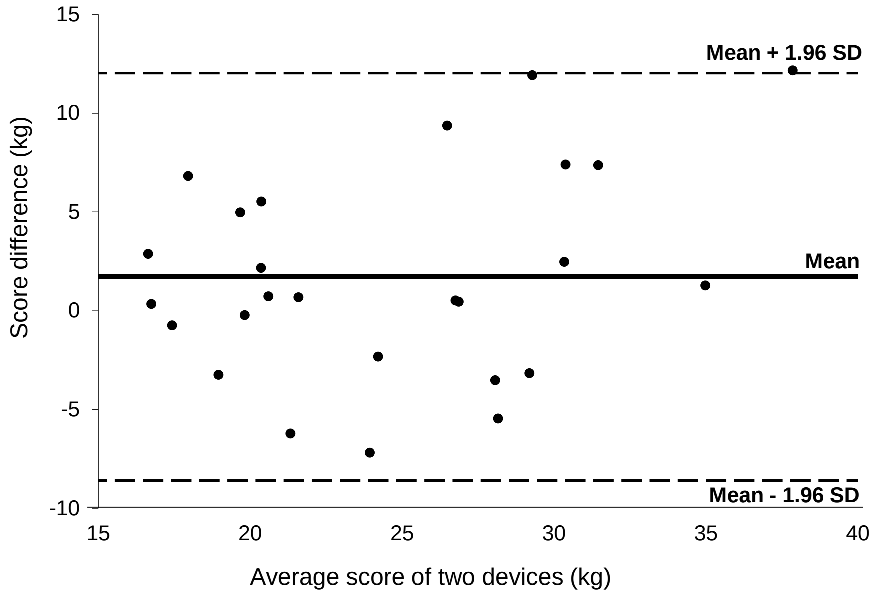

Assessor-B ankle dorsiflexors:

(n: validity 25; Lafayette intra-rater reliability 28; inter-device reliability 27)

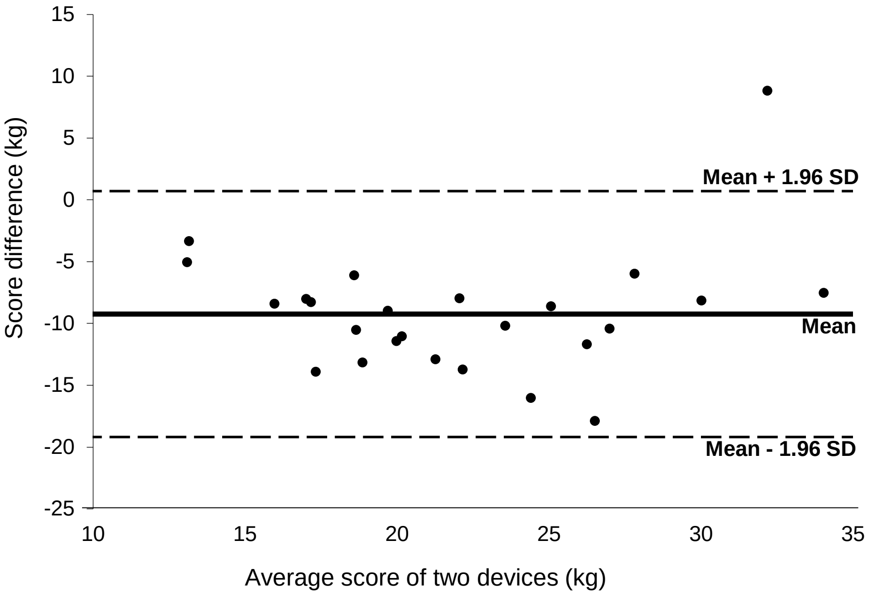

Assessor-B ankle plantarflexors:

(n: validity 25; Lafayette intra-rater reliability 28; inter-device reliability 27)

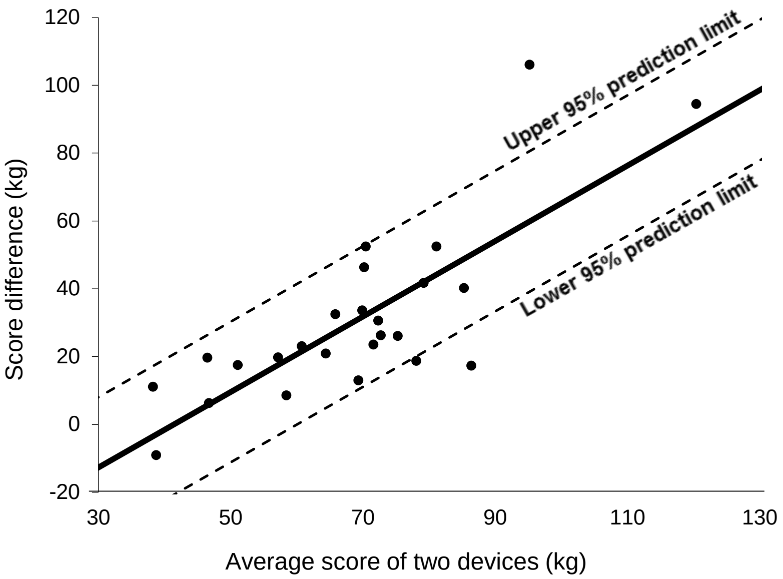

R = 0.79; $\mathbf{R^2}$ **=** 0.63; **Slope =** 1.11; **Intercept =** -45.97

Assessor-B hip abductors:

(n: validity 26; Lafayette intra-rater reliability 29; inter-device reliability 28)

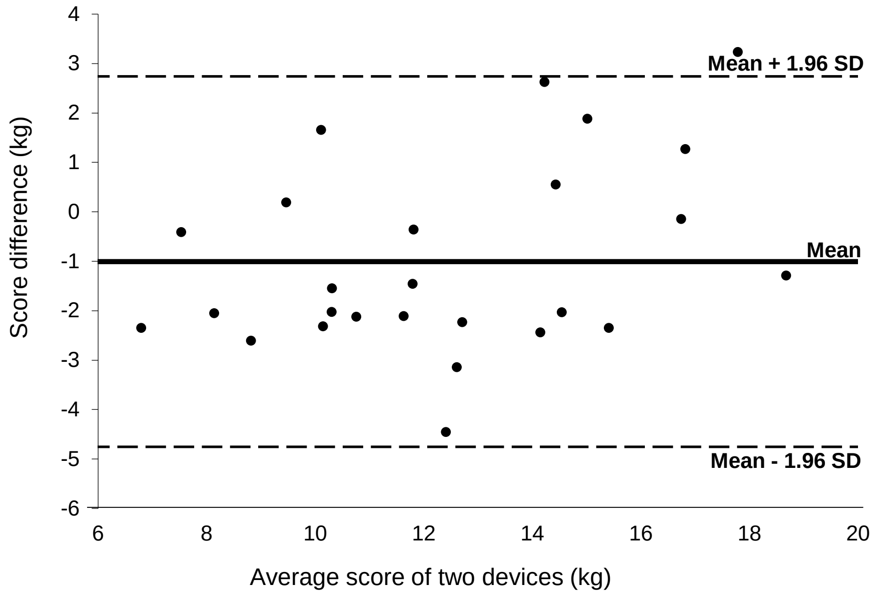

Assessor-B hip adductors:

(n: validity 24; Lafayette intra-rater reliability 27; inter-device reliability 26)

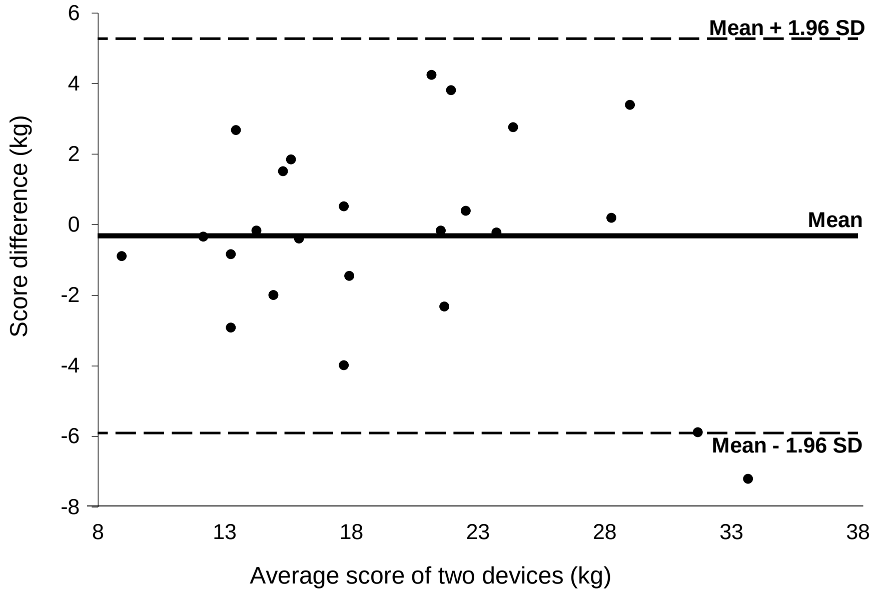

Assessor-B hip extensors:

(n: validity 25; Lafayette intra-rater reliability 28; inter-device reliability 27)

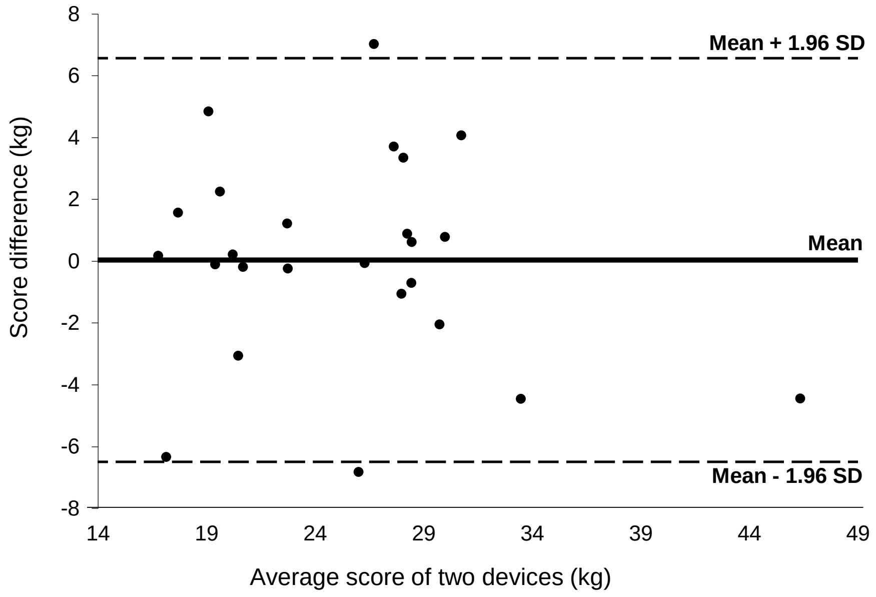

Assessor-B hip flexors:

(n: validity 26; Lafayette intra-rater reliability 29; inter-device reliability 27)

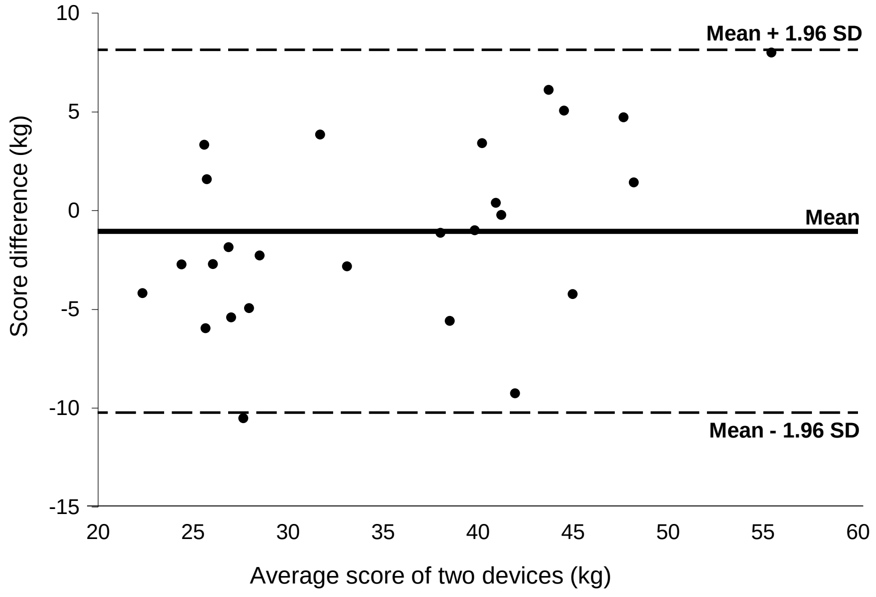

Assessor-B knee extensors:
(n: validity 25; Lafayette intra-rater reliability 28; inter-device reliability 28)

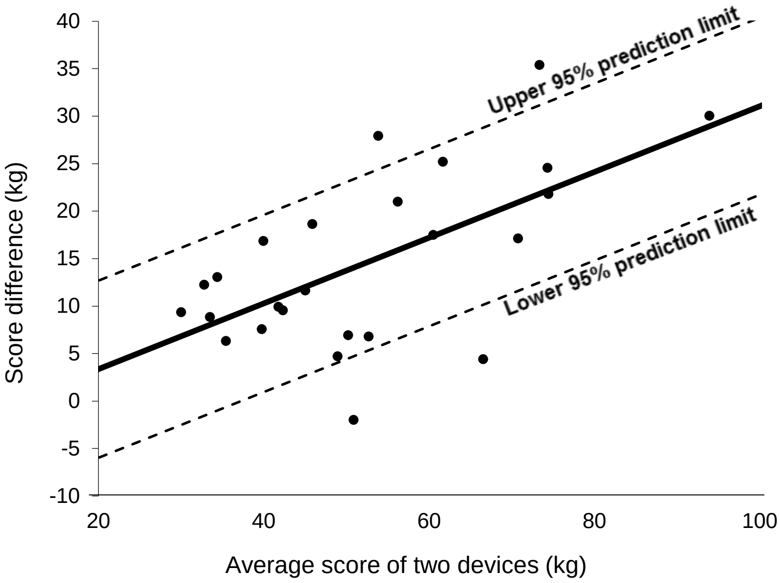

R = 0.61**; R^2 =** 0.37**; Slope =** 0.35**; Intercept =** -3.55

Assessor-B knee flexors:
(n: validity 26; Lafayette intra-rater reliability 29; inter-device reliability 24)

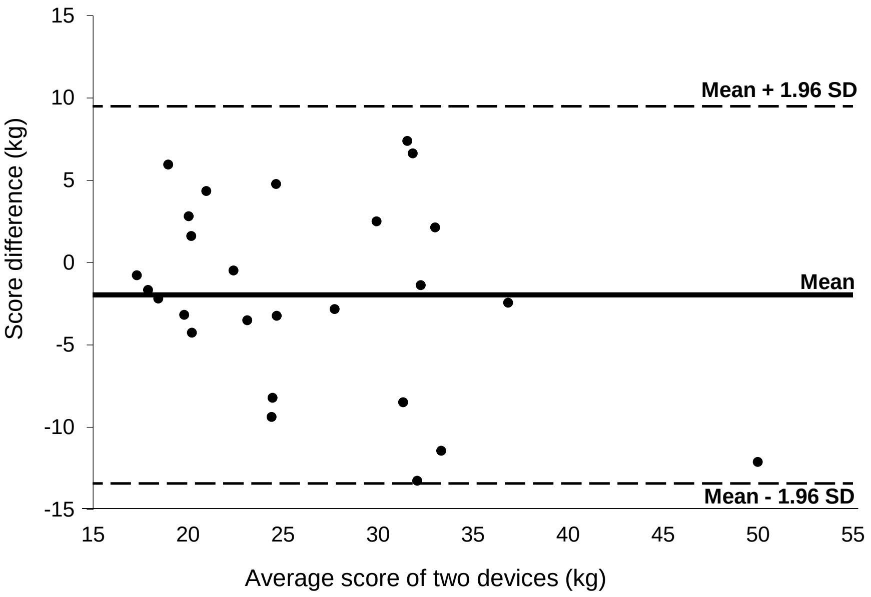

Supplement: S1 File — (PDF) [file pone.0140822.s001.pdf]
