# Supplementary material for: Assessment of Lower Limb Muscle Strength and Power Using Hand-Held and Fixed Dynamometry: A Reliability and Validity Study
Source: PLoS One. 2015 Oct 28;10(10):e0140822. doi: 10.1371/journal.pone.0140822 (PMC4624940; doi:10.1371/journal.pone.0140822)

Assessment of lower limb muscle strength and power using hand-held and fixed dynamometry: a reliability and validity study

S4 Appendix – Bland-Altman Plots indicating the validity of the Hoggan HHD in comparison with the KinCom for analysis of rate of force development (kg/s)

Note: Not all reliability and validity analyses include 30 data sets. Some participants mentioned soreness in some muscle groups unrelated to the testing procedures, and as such those sore muscles were not tested. The knee extensors and ankle plantarflexors of one participant were unable to be tested due to high strength and power levels of the participant. One participant was unable to attend the second testing session. The KinCom was unable to be used at all for four participants as the device was being repaired and five participants only had one session of KinCom data collection. On many occasions the Hoggan software failed to save the raw data during testing, resulting in fewer data sets for all analyses involving the Hoggan device. The parentheses prior to each figure details the number of participants that were used for analysis.

Assessor-A ankle dorsiflexors:

(n: validity 25; Hoggan intra-rater reliability 25; inter-rater reliability 26)

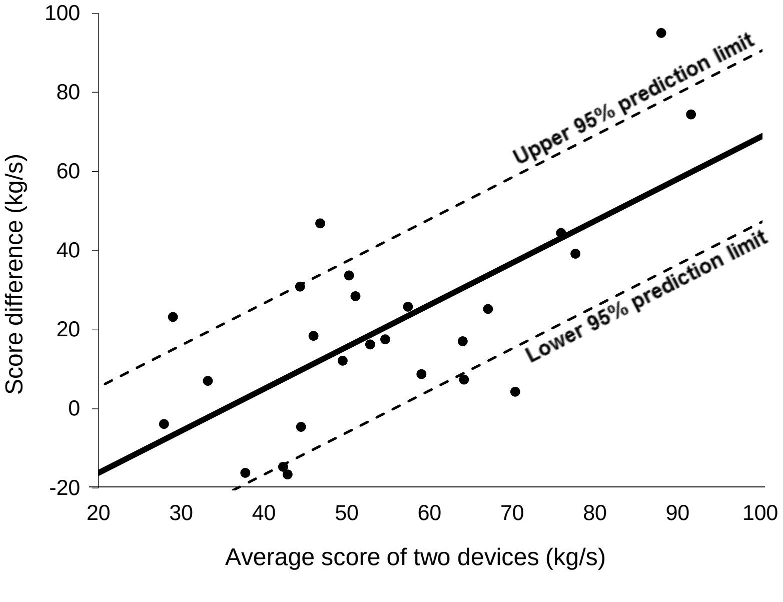

R = 0.69**; R^2 =** 0.47**; Slope =** 1.06**; Intercept =** -37.36

Assessor-A ankle plantarflexors:

(n: validity 24; Hoggan intra-rater reliability 23; inter-rater reliability 26)

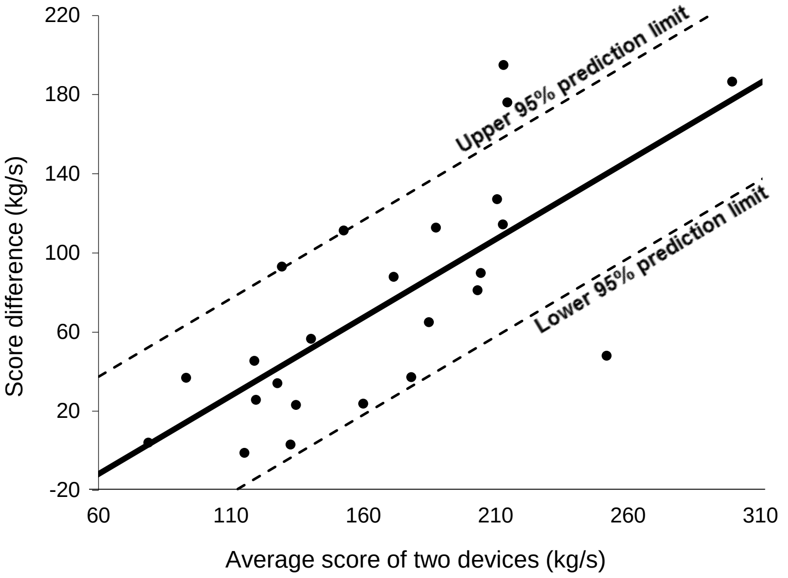

R = 0.73**; R^2 =** 0.53**; Slope =** 0.79**; Intercept =** -59.08

Assessor-A hip abductors:

(n: validity 25; Hoggan intra-rater reliability 26; inter-rater reliability 28)

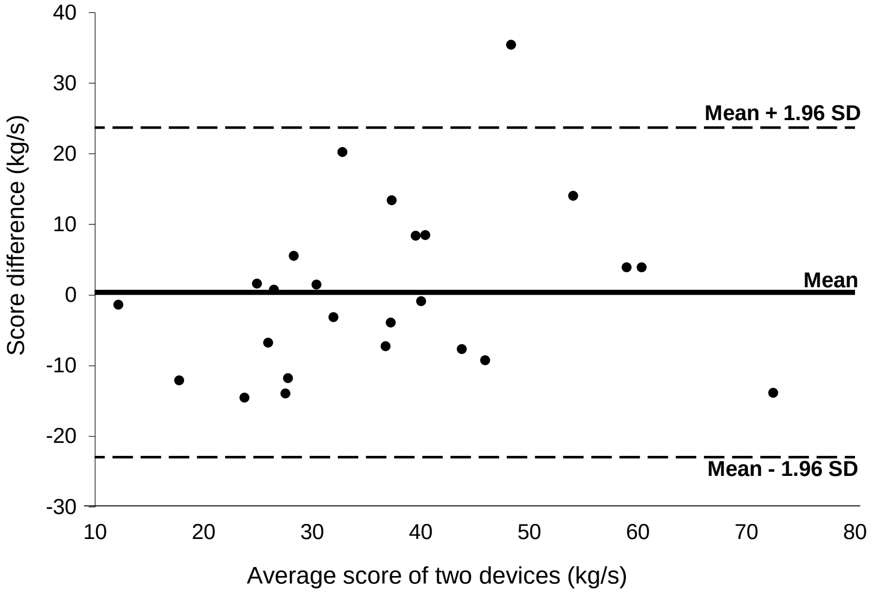

Assessor-A hip adductors:

(n: validity 20; Hoggan intra-rater reliability 18; inter-rater reliability 23)

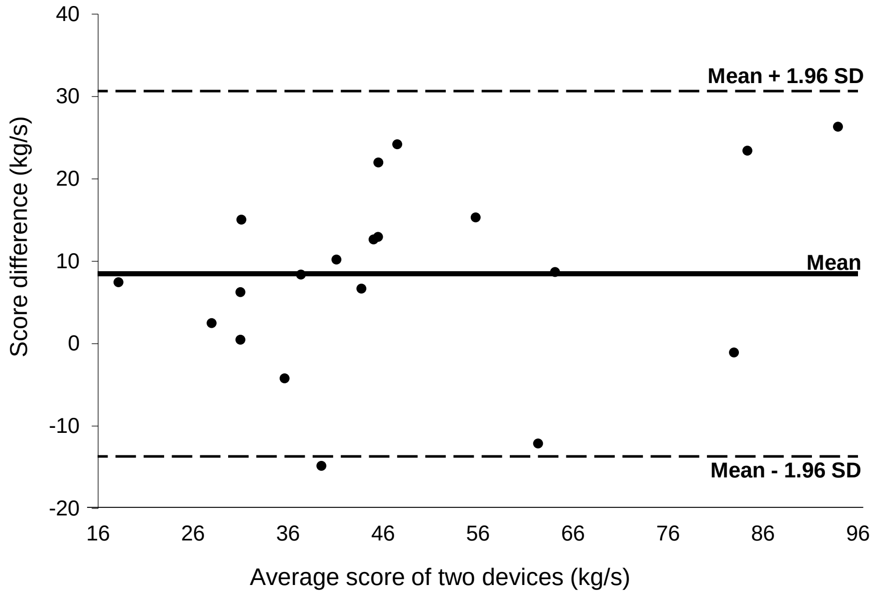

Assessor-A hip extensors:

(n: validity 25; Hoggan intra-rater reliability 26; inter-rater reliability 27)

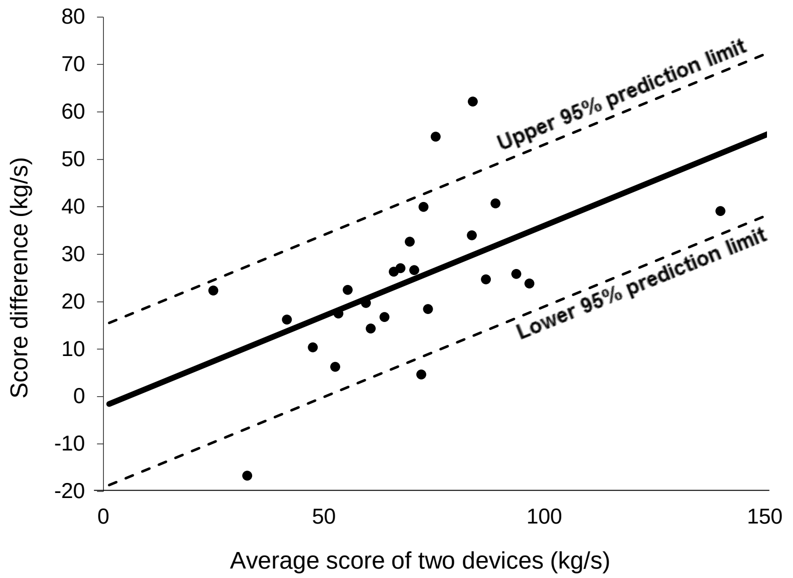

R = 0.55; **R^2 =** 0.30; **Slope =** 0.38; **Intercept =** -2.06

Assessor-A hip flexors:

(n: validity 23; Hoggan intra-rater reliability 23; inter-rater reliability 23)

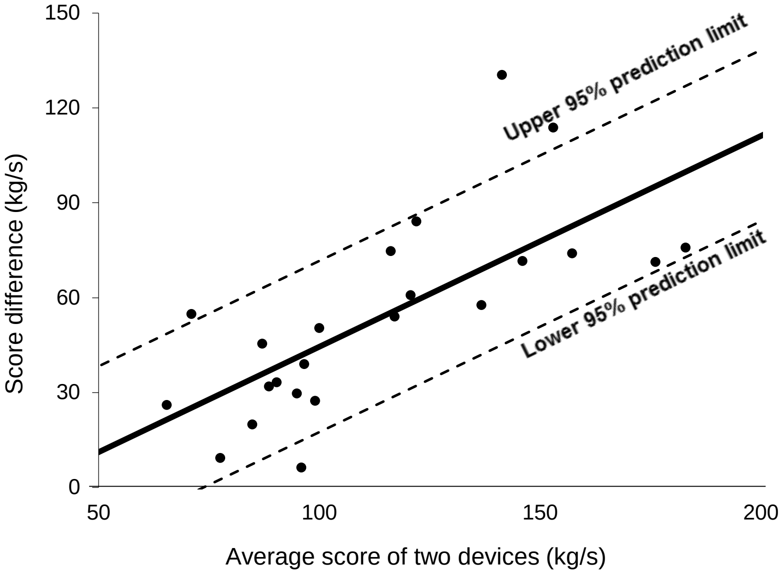

R = 0.71**;** $\mathbf{R^2}$ **=** 0.51**; Slope =** 0.67**; Intercept =** -22.08

Assessor-A knee extensors:

(n: validity 24; Hoggan intra-rater reliability 20; inter-rater reliability 25)

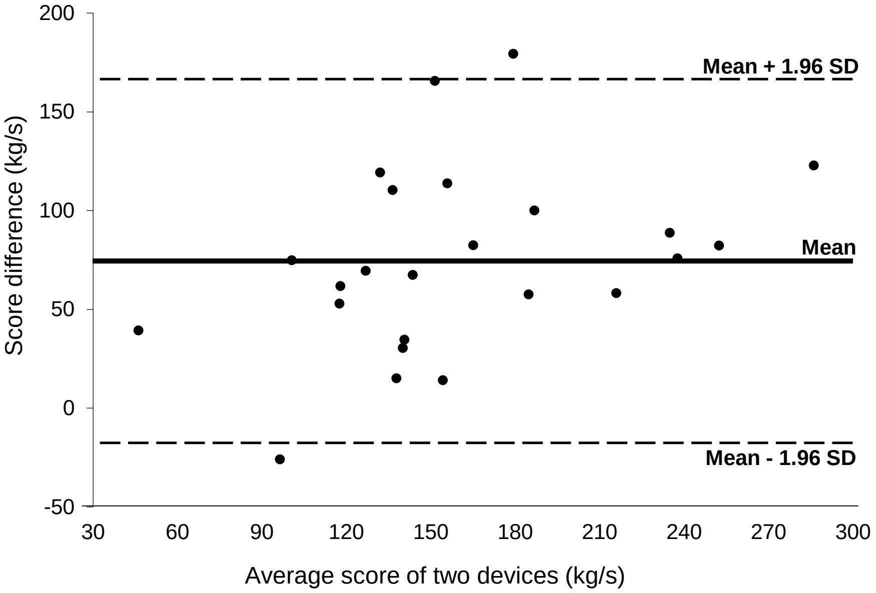

Assessor-A knee flexors:

(n: validity 17; Hoggan intra-rater reliability 17; inter-rater reliability 16)

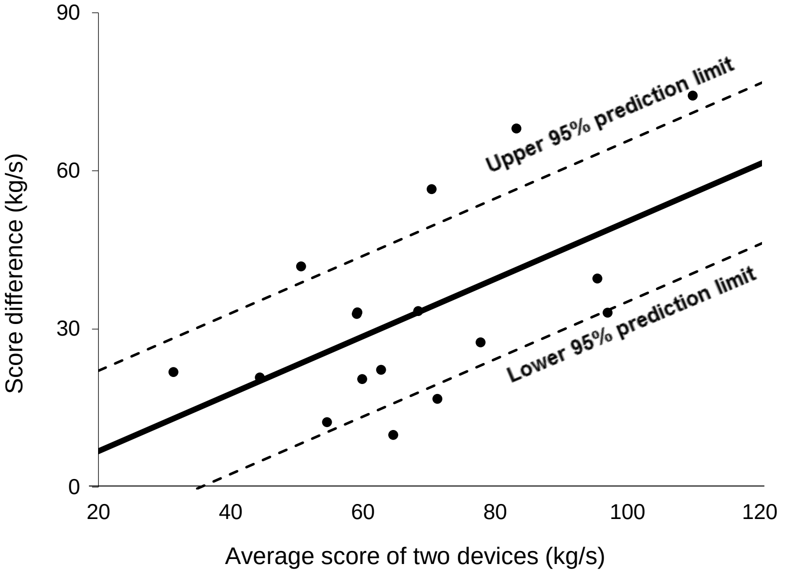

R = 0.59**; R^2 =** 0.35**; Slope =** 0.54**; Intercept =** -4.03

Assessor-B ankle dorsiflexors:

(n: validity 25; Hoggan intra-rater reliability 27)

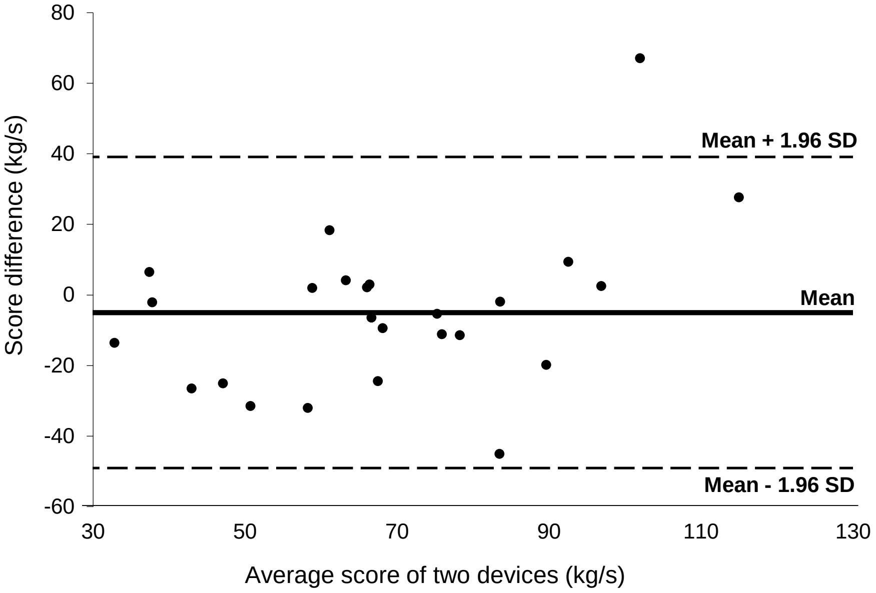

Assessor-B ankle plantarflexors:

(n: validity 24; Hoggan intra-rater reliability 23)

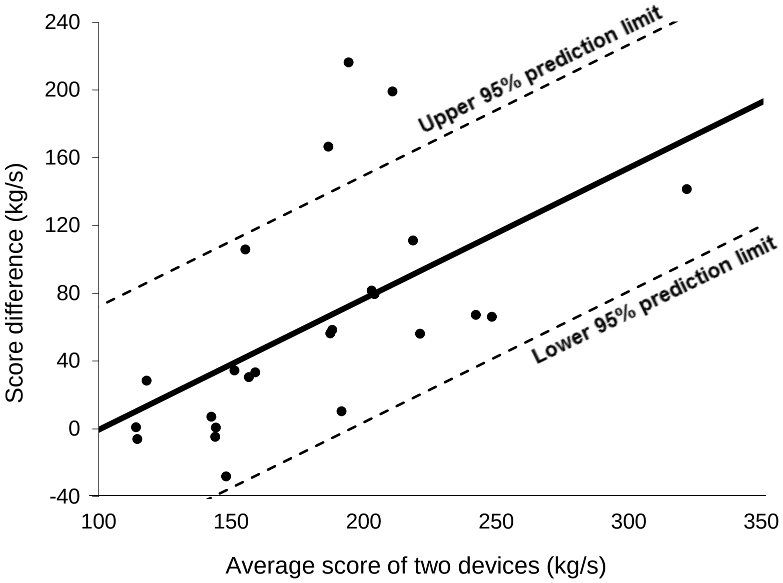

R = 0.57**;** $\mathbf{R^2}$ **=** 0.33**; Slope =** 0.77**; Intercept =** -77.81

Assessor-B hip abductors:

(n: validity 26; Hoggan intra-rater reliability 28)

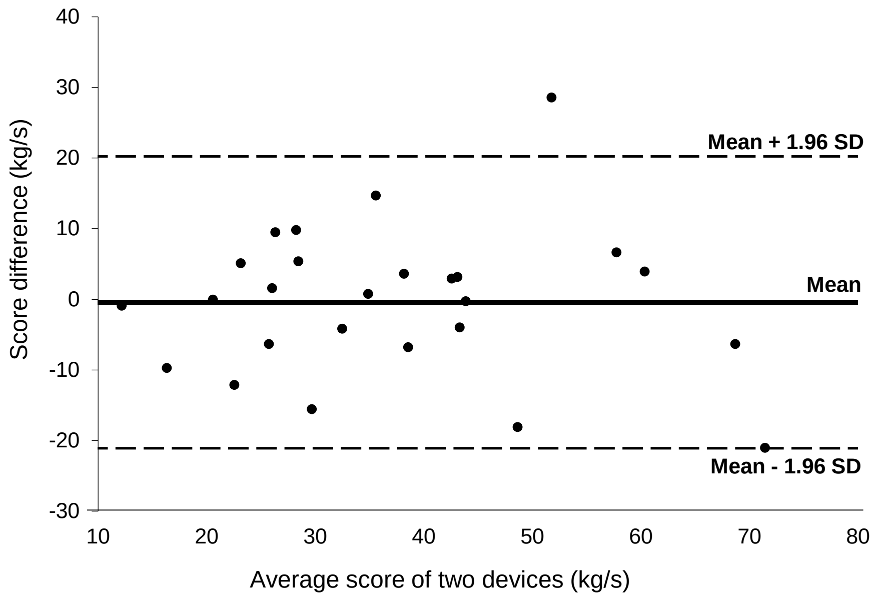

Assessor-B hip adductors:

(n: validity 23; Hoggan intra-rater reliability 22)

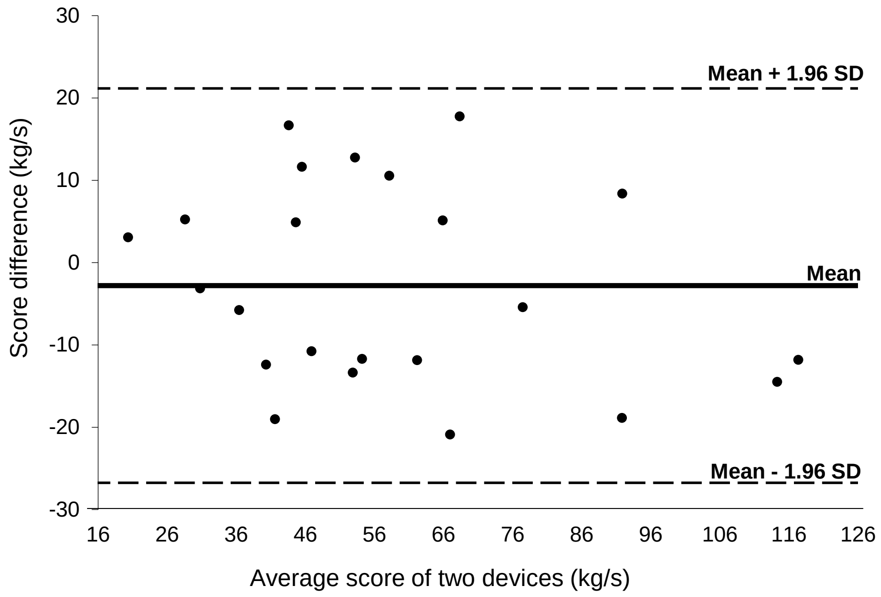

Assessor-B hip extensors:

(n: validity 24; Hoggan intra-rater reliability 24)

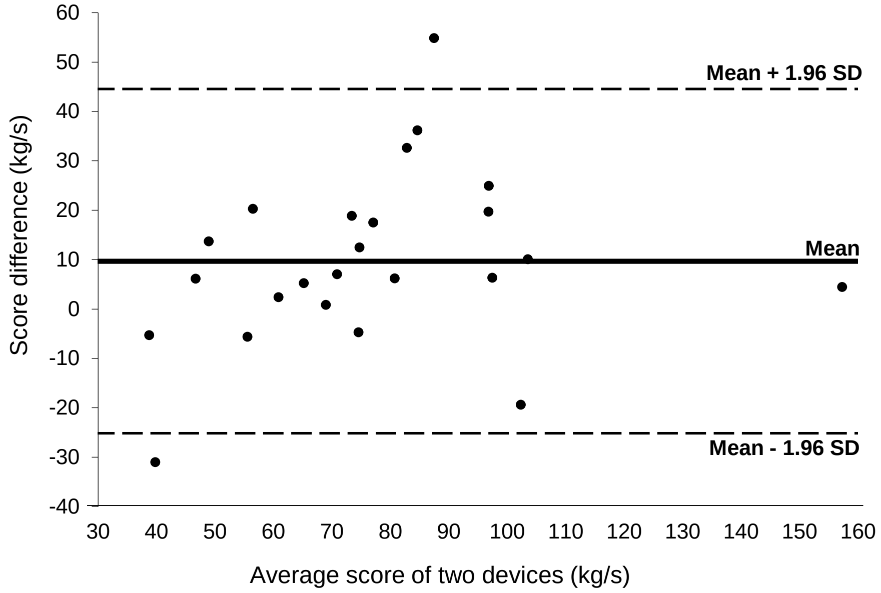

Assessor-B hip flexors:

(n: validity 24; Hoggan intra-rater reliability 25)

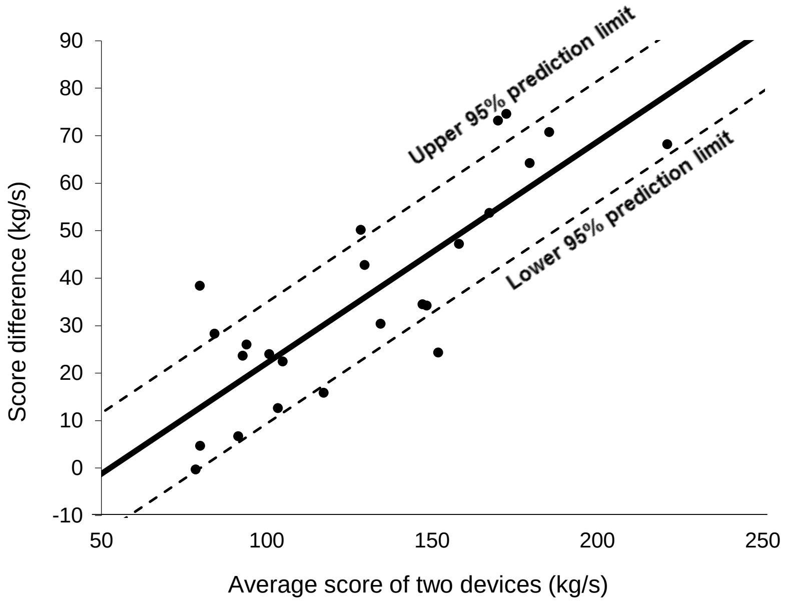

R = 0.83**; R^2 =** 0.70**; Slope =** 0.47**; Intercept =** -24.44

Assessor-B knee extensors:
(n: validity 25; Hoggan intra-rater reliability 27)

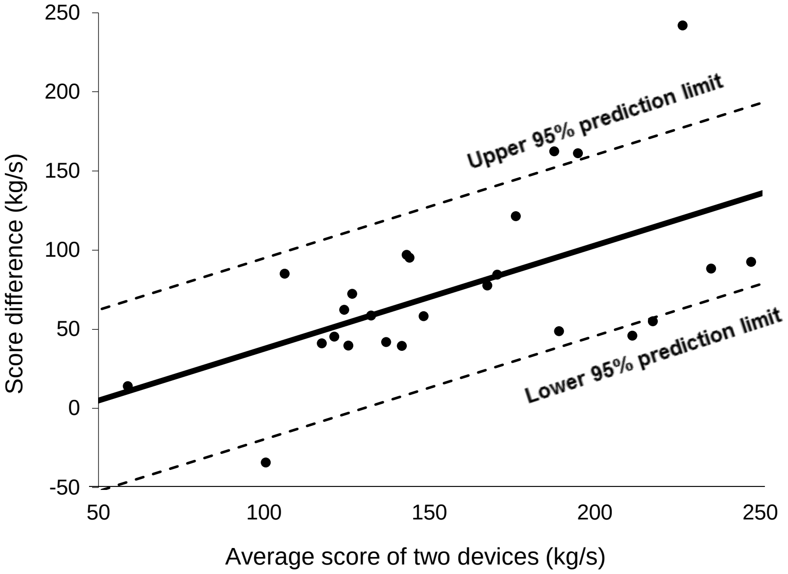

R = 0.56**; R^2 =** 0.32**; Slope =** 0.65**; Intercept =** -27.60

Assessor-B knee flexors:
(n: validity 21; Hoggan intra-rater reliability 19)

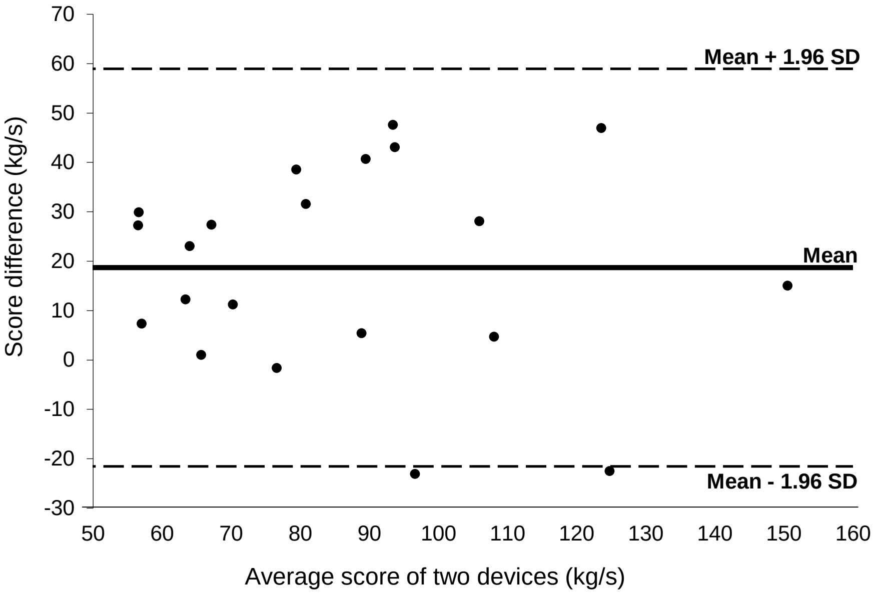

Supplement: S4 File — (PDF) [file pone.0140822.s004.pdf]
